# Improved detection limits of *J*‐coupled neurometabolites in the human brain at 7 T with a *J*‐refocused sLASER sequence

**DOI:** 10.1002/nbm.4801

**Published:** 2022-08-10

**Authors:** Chloé Najac, Vincent O. Boer, Hermien E. Kan, Andrew G. Webb, Itamar Ronen

**Affiliations:** ^1^ C. J. Gorter Center for High Field MRI, Department of Radiology Leiden University Medical Center Leiden The Netherlands; ^2^ Danish Research Centre for Magnetic Resonance Copenhagen University Hospital Hvidovre Hvidovre Denmark

**Keywords:** 7 T, aspartate, detection limits, glutamate, glutamine, human brain, *J*‐coupled metabolites, *J*‐refocused sLASER

## Abstract

In a standard spin echo, the time evolution due to homonuclear couplings is not reversed, leading to echo time (TE)‐dependent modulation of the signal amplitude and signal loss in the case of overlapping multiplet resonances. This has an adverse effect on quantification of several important metabolites such as glutamate and glutamine. Here, we propose a *J*‐refocused variant of the sLASER sequence (*J*‐sLASER) to improve quantification of *J*‐coupled metabolites at ultrahigh field (UHF). The use of the sLASER sequence is particularly advantageous at UHF as it minimizes chemical shift displacement error and results in relatively homogenous refocusing. We simulated the MRS signal from brain metabolites over a broad range of TE values with sLASER and *J*‐sLASER, and showed that the signal of *J*‐coupled metabolites was increased with *J*‐sLASER with TE values up to ~80 ms. We further simulated “brain‐like” spectra with both sequences at the shortest TE available on our scanner. We showed that, despite the slightly longer TE, the *J*‐sLASER sequence results in significantly lower Cramer–Rao lower bounds (CRLBs) for *J*‐coupled metabolites compared with those obtained with sLASER. Following phantom validation, we acquired spectra from two brain regions in 10 healthy volunteers (age 38 ± 15 years) using both sequences. We showed that using *J*‐sLASER results in a decrease of CRLBs for *J*‐coupled metabolites. In particular, we measured a robust ~38% decrease in the mean CRLB (glutamine) in parietal white matter and posterior cingulate cortex (PCC). We further showed, in 10 additional healthy volunteers (age 34 ± 15 years), that metabolite quantification following two separate acquisitions with *J*‐sLASER in the PCC was repeatable. The improvement in quantification of glutamine may in turn improve the independent quantification of glutamate, the main excitatory neurotransmitter in the brain, and will simultaneously help to track possible modulations of glutamine, which is a key player in the glutamatergic cycle in astrocytes.

## Graphical abstract

We propose a *J*‐refocused variant of the sLASER sequence (*J*‐sLASER) that improves the detection limits of *J*‐coupled metabolites at ultrahigh field in the human brain. Following simulation and validation in phantom, we show significant enhancement in the detection of glutamine, glutamate, and aspartate. This sequence could significantly improve the detection limits of small changes in diseases or during functional MRS experiments.

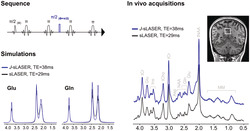

**Abbreviations used**
Asp: aspartate
BW: bandwidth
CoV: coefficient of variation
Cr: creatine
CRLB: Cramer–Rao lower bound
CSDE: chemical shift displacement error
CSF: cerebrospinal fluid
fMRS: functional MRS
FOCI: frequency offset corrected inversion
FWHM: full width at half maximum
GABA: gamma‐aminobutyric acid
Gln: glutamine
Glu: glutamate
GM: gray matter
GPC: glycerophophorylcholine
GSH: glutathione
Ins: myo‐inositol
Lac: lactate
MM: macromolecule
NAA: N‐acetyl‐aspartate
NAAG: N‐acetyl‐aspartate glutamate
np: number of points
NSA: number of spectral acquisitions
PCC: posterior cingulate cortex
PCho: phosphocholine
PCr: phosphocreatine
PE: phosphoethanolamine
PRESS: point‐resolved spectroscopy
PWM: parietal white matter
sIns: scyllo‐inositol
sLASER: semi‐LASER
SNR: signal‐to‐noise ratio
STEAM: stimulated echo acquisition mode
STRESS: STEAM+PRESS
Tau: taurine
UHF: ultrahigh field
VAPOR: variable power and optimized relaxation delays
VOI: volume of interest
WM: white matter

## 1 INTRODUCTION

Proton magnetic resonance spectroscopy (^1^H MRS) offers a specific insight on physiology and metabolism in healthy and diseased tissues in vivo.^1^, ^2^, ^3^, ^4^ ^1^H MRS provides information on cellular processes, as metabolites of interest are primarily located in the intracellular space of neurons and glia. As some metabolites such as glutamate (Glu) and N‐acetyl aspartate (NAA) are mainly found in neurons, whereas others, such as glutamine (Gln) and myo‐inositol (Ins) are mostly concentrated in glia,^5^ ^1^H MRS offers the possibility of investigating cellular processes in a cell‐specific manner.

With the advent of MR scanners operating at ultrahigh field (UHF, B_0_ ≥ 7 T), quantification of metabolite concentration is greatly enhanced due to both improved sensitivity and spectral resolution.^1^ The spectra of several neurometabolites of interest, such as Glu, Gln, and lactate (Lac), however, suffer from loss of signal due to the modulation of *J*‐coupled resonances during the echo time (TE),^2^, ^6^, ^7^ as localized MRS relies primarily on spin echo and stimulated echo sequences.^8^, ^9^ While the spin–spin coupling is field‐independent, the increase in spectral linewidth at higher magnetic field further contributes to the degradation of signal originating from *J*‐coupled spins. MRS acquisition at short TEs is thus preferred as it limits signal loss due to *J*‐modulation and thus maximizes signal‐to‐noise ratio (SNR) for metabolites with *J*‐coupled spins.^2^

Single‐voxel MRS with optimized TE, or editing techniques based on *J*‐refocusing, have been proposed to improve the detection limits of specific *J*‐coupled metabolites of interest, such as gamma‐aminobutyric acid (GABA), glutathione (GSH), Lac, Glu, Gln, and 2‐hydroxyglutarate.^1^, ^10^, ^11^, ^12^, ^13^, ^14^, ^15^, ^16^, ^17^, ^18^, ^19^ Such sequences require a relatively long TE, resulting in lower SNR compared with standard sequences with short TE, and there is no consensus regarding the relative accuracy in quantification of editing and optimized TE sequences compared with conventional MRS with short TE values.^20^, ^21^, ^22^

It was independently shown by Takegoshi et al. and van Zijl et al. that the evolution of *J*‐coupling in weakly coupled spins (I = 1/2) can be fully or partially refocused when a π/2 RF pulse with phase perpendicular to the excitation pulse is added at the time of the first echo formation in a double spin‐echo sequence.^23^, ^24^ Such *J*‐refocused spin‐echo sequences have been implemented and used in a variety of in vitro and in vivo MRS applications,^23^, ^24^, ^25^, ^26^, ^27^, ^28^, ^29^, ^30^, ^31^, ^32^ and were successfully implemented in a PRESS sequence and used in MRS imaging of the human brain at 4 and 7 T.^33^, ^34^ These studies illustrated the potential of *J*‐refocused sequences in improving quantification of fatty acids in calf bone marrow or skeletal muscle,^26^ citrate in prostate cancer,^30^ and Glu/Gln in the brain.^28^, ^33^, ^34^ An additional approach for improving detection limits of both *J*‐coupled and non–*J*‐coupled metabolites using a PRESS/STEAM hybrid (STRESS) was also suggested.^35^, ^36^ These approaches are all PRESS‐based sequences, limiting their applications at UHF because PRESS sequences suffer from significant chemical shift displacement errors (CSDEs) due to the relatively small bandwidth (BW) of the two refocusing pulses.^37^ The use of nonadiabatic refocusing pulses in the PRESS sequence also results in strong sensitivity to transmit RF field (B_1_^+^) inhomogeneity, in turn resulting in imperfect refocusing and additional signal loss. As an alternative spin echo‐based sequence, the sLASER sequence uses two pairs of high‐BW adiabatic refocusing pulses, thus offering a significantly smaller CSDE and more homogeneous refocusing within the volume of interest (VOI). This leads to improved spectral quality and reliable quantification of low concentration *J*‐coupled metabolites at a reasonably short TE, typically about 30 ms^2^. Finally, the succession of adiabatic π RF pulse of the sLASER (or LASER) sequence emulates a Carr–Purcell train, which also minimizes the *J*‐evolution for *J*‐coupled metabolites.^38^, ^39^ The sLASER sequence has thus been recommended for spin echo‐based acquisitions at UHF (B_0_ ≥ 7 T).^4^

We recently proposed a proof‐of‐principle for the *J*‐refocused variant of the sLASER sequence based on the perfect echo sequence,^40^ which we termed *J*‐sLASER. This sequence was subsequently illustrated in a preclinical setup.^41^ Here, we demonstrate that *J*‐sLASER provides significant recovery of the signal from *J*‐coupled metabolites in the human brain at 7 T. We validate the *J*‐sLASER sequence with simulations and phantom acquisitions at 7 T and we compare its performance with the standard sLASER sequence. We then use *J*‐sLASER to acquire data from the posterior cingulate cortex (PCC) and parietal white matter (PWM) in the human brain at 7 T, illustrating the improvement in quantification of *J*‐coupled metabolites, in particular Gln, compared with the standard sLASER sequence. Finally, we show results from a repeatability study, in which test–retest sLASER and *J*‐sLASER acquisitions in the PCC were performed to examine the stability of metabolite quantification following measurement with both sequences.

## 2 MATERIALS AND METHODS

### 2.1 Pulse sequences

#### 2.1.1 Standard sLASER

**FIGURE 1:**
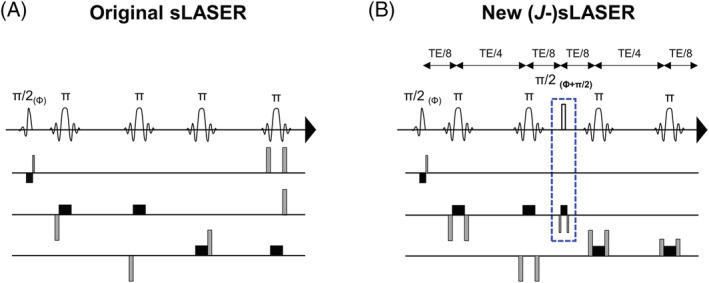
Diagrams of the (A) sLASER and (B) *J*‐sLASER sequences. Slice selection gradients are represented in black and refocusing/spoiler gradients are shown in gray. The crusher gradient scheme for the *J*‐sLASER sequence was modified from its original form to ensure fully refocused transverse magnetization at the time of the π/2 pulse. All π pulses are adiabatic slice‐selective FOCI RF pulses. The additional π/2 square RF pulse with concomitant slice selection and refocusing gradients in the *J*‐sLASER sequence are highlighted with a dotted blue rectangle.

A sLASER sequence with an asymmetric sinc excitation pulse and four adiabatic slice‐selective FOCI pulses^42^ was used. As illustrated in Figure 1A and previously published,^43^ a combination of spoiler gradients surrounding the FOCI pulses was used to minimize artifacts from unwanted spurious echoes while minimizing TE. With a transmit RF field B_1_ = 18 μT, the minimum TE achievable with the sLASER sequence was 29 ms on our 7 T MR clinical scanner.

#### 2.1.2 *J*‐sLASER sequence

The standard sLASER sequence was modified to have equidistant echo‐spacing and to incorporate a π/2 RF pulse and concomitant slice selection and refocusing gradients at the time of the second echo (Figure 1B). The phase of the added π/2 pulse was set to be orthogonal to that of the π/2 excitation pulse, as dictated by the “perfect echo” condition. We used a square pulse (pulse duration = 0.33 ms, BW = 3063 Hz), as this made it possible to obtain the required BW while using the shortest pulse available, thus minimizing the time added to the shortest TE of the original sLASER sequence. We opted for making this added module slice selective, thereby paying a small price in minimum TE, to minimize out‐of‐volume excitation to one slice rather than generating newly excited transverse magnetization from the entire volume. It is important to note that to avoid generation of a stimulated echo and subsequent loss of 50% of the signal, the crusher scheme of the original sLASER sequence had to be modified so that the transverse magnetization was fully refocused at the time of the additional π/2 pulse. Finally, an eight‐step phase cycling scheme (phase of the first π/2 RF pulse and receiver: 0, 0, 90, 90, 180, 180, 270, 270; phase of the second π/2 and all π RF pulses: 90, 270, 180, 0, 270, 90, 0, 180) was used for additional suppression of unwanted coherence pathways and an outer‐volume suppression module was used to reduce contamination. We arranged the phase‐cycling order so that the phase of the “perfect echo” π/2 RF pulse is cycled between opposite values within consecutives pairs of acquisitions, which minimizes the effect of any head motion between individual acquisitions on the final signal summation. With B_1_ = 18 μT, the minimum TE achievable with the *J*‐sLASER sequence was 38 ms on our 7 T MR clinical scanner.

### 2.2 Hardware

All experiments were conducted on a Philips 7 T whole‐body MRI scanner (Philips Healthcare, The Netherlands) equipped with a quadrature transmit/32‐channel receive head coil (Nova Medical, USA) and gradient coils with a maximum gradient strength of 40 mT/m and a slew rate of 200 T/m/s.

### 2.3 Simulations

#### 2.3.1 General

Simulated spectra of brain metabolites were generated using in‐house Matlab (MathWorks, Natick, MA, USA) programs^44^ based on the density matrix formalism, which included known chemical shifts and *J*‐coupling constants of the proton resonances of the metabolites, to calculate the time evolution of the signal for the two pulse sequences. For simplicity, we assumed impulse RF pulses (i.e., without any time evolution during the pulse) in both sequences. Proton chemical shifts and *J*‐coupling constant values were taken from Govindaraju et al.^46^ and T_2_ relaxation values were based on previously measured values.^47^, ^48^

#### 2.3.2 Time evolution of the signal of selected *J*‐coupled resonances under sLASER and *J*‐sLASER

To evaluate the effect of *J*‐evolution on the signal of different metabolites, we simulated for both sequences spectra of selected metabolites that contain *J*‐coupled protons within a range of TE values from 10 to 200 ms. Signal amplitudes were calculated as integral under the peaks of the *J*‐coupled resonances, and were normalized against the sLASER signal at the shortest TE in our simulations (TE_min_ = 10 ms). Finally, we investigated the evolution of the normalized signal for both sequences as a function of TE.

#### 2.3.3 Monte Carlo simulation of brain‐like spectra with sLASER and *J*‐sLASER

To estimate the performance of both sequences in realistic conditions, we performed a Monte Carlo simulation based on brain‐like spectra. The simulated datasets consisted of a weighted sum of signals of 17 metabolites. Relative metabolite concentrations were based on published in vivo concentration values^49^, ^50^, ^51^, ^52^: alanine (Ala, 0.5 mM), aspartate (Asp, 2 mM), creatine (Cr, 5 mM), GABA (1 mM), Gln (3 mM), Glu (10 mM), glycerophophorylcholine (GPC, 0.5 mM), GSH (1 mM), myo‐inositol (Ins, 7 mM), Lac (0.5 mM), NAA (12 mM), N‐acetylaspartylglutamate (NAAG, 1 mM), phosphocholine (PCho, 0.5 mM), phosphocreatine (PCr, 5 mM), phosphorylethanolamine (PE, 1.5 mM), scyllo‐inositol (sIns, 0.5 mM), and taurine (Tau, 1.5 mM). The TE values used for the simulation were the shortest achievable TE values on our scanner for both sequences: TE = 29 ms for sLASER and TE = 38 ms for *J*‐sLASER. A measured macromolecular (MM) baseline was also added to the simulated spectra. The amplitude of MM was set based on the ratio of the MM peak at 0.9 ppm to that of the total creatine (tCr) singlet at 3.02 ppm observed in in vivo acquisitions. A separate water spectrum (concentration of 40 mM) was also generated as a reference dataset. Line‐broadening and Gaussian noise were added to reach relevant Cramer–Rao lower bound (CRLB) values for the main metabolites when compared with the literature and similar SNR and full width at half maximum (FWHM) values for both sequences (SNR 104 ± 5 for sLASER at TE = 29 ms, 108 ± 5 for sLASER at TE = 38 ms, and 108 ± 6 for *J*‐sLASER at TE = 38 ms; FWHM 9.2 ± 0.5 Hz for sLASER at TE = 29 ms and *J*‐sLASER at TE = 38 ms and 9.1 ± 0.4 Hz for sLASER at 38 ms). This procedure was repeated 1000 times with different random noise realizations for both sequences, yielding 1000 different concentrations and CRLB values for each metabolite and condition. The output was then broken into 50 subgroups to evaluate the distributions of CRLB values within 20 iterations and to calculate the coefficient of variation (CoV) of CRLB values across subgroups. The CoV is defined as the ratio of the standard deviation σ to the mean μ.

### 2.4 Phantom experiments

In vitro sequence validation was subsequently performed on a MRS “Braino” phantom (GE Medical Systems, Milwaukee, WI, USA^53^) that contained NAA (12.5 mM), Cr (10 mM), choline (Cho; 3 mM), Ins (7.5 mM), L‐glutamic acid (12.5 mM), DL‐lactic acid (5 mM), sodium azide (0.1%), 50 mM of potassium phosphate monobasic [KH_2_PO_4_], 56 mM of sodium hydroxide [NaOH], and 1 mL/L of Gd‐DPTA solution (Magnevist). MRS data were acquired with sLASER and *J*‐sLASER using a range of TE values between 38 and 148 ms, TR = 5000 ms, number of signal averaged (NSA) = 8, BW = 3 kHz, and the number of time domain points (np) = 2048.

### 2.5 In vivo experiments

A total of 20 healthy volunteers (age 34 ± 15 years, 15 females and five males) participated in this study. The study adhered to the guidelines of the Leiden University Medical Center Institutional Review Board (The Netherlands). Written informed consent was obtained from all subjects prior to the study. In all experiments, a 3D T_1_W gradient‐echo acquisition (TR/TE = 5/2 ms, flip angle 7°, resolution 1 mm isotropic) was used for planning the MRS experiments.

#### 2.5.1 Cross‐sectional study

Data were acquired in 10 healthy volunteers (age 38 ± 15 years, seven females and three males). A 2 x 2 x 2 cm^3^ VOI was positioned in the PCC (n = 10) and PWM (n = 10). In both VOIs, spectra were acquired with (1) sLASER at the shortest achievable TE of 29 ms; (2) *J*‐sLASER at the shortest achievable TE of 38 ms; and (3) sLASER at TE = 38 ms. All spectra were acquired with TR = 6000 ms, NSA = 48, BW = 3 kHz, and np = 1024. Water suppression was achieved using the variable pulse power and optimization relaxation delays (VAPOR) sequence.^54^ Outer‐volume‐suppression was also performed using a total of 10 saturation bands positioned circularly around the VOI. The bands (REST slabs) were interleaved with the water suppression. Water data were acquired using identical parameters, without water suppression and with NSA = 8. VOI‐localized B_0_ shimming up to second order (pencil‐beam approach, Philips product tool) was performed.

#### 2.5.2 Repeatability study

Data were acquired in 10 healthy volunteers (age 31 ± 15 years, eight females and two males) in two consecutive sessions. A 2 x 2 x 2 cm^3^ VOI was positioned in the PCC, as described above. Spectra were acquired with sLASER and with *J*‐sLASER using the same parameters as above. Subjects were taken out of the scanner, repositioned and scanned a second time with the same protocol and the VOI was in the same position. Within‐subject CoVs for metabolite level were estimated as described in de Boer et al.^55^

### 2.6 Postprocessing

#### 2.6.1 MRI

T_1_‐weighted images were segmented into probabilistic tissue maps for white matter (WM), gray matter (GM), and cerebrospinal fluid (CSF) using FSL (Brain extraction Tool^56^ and FAST^57^ algorithm in the FMRIB Software Library). An in‐house Matlab routine was then used to create binary tissue maps (values are either 0 or 1, with 1 given to the tissue with highest probabilistic value) and to quantify the volume of each tissue type within each VOI.

#### 2.6.2 MRS

In vivo and in vitro spectra were corrected for eddy currents using nonwater‐suppressed data using an in‐house Matlab routine. The phase‐cycling order was such that individual in vivo metabolites spectra were first summed pairwise to suppress the major contribution of out‐of‐volume lipids to the spectra. Subsequently, spectra were corrected for phase and frequency drifts using an in‐house Matlab routine based on spectral registration^58^ performed in the frequency domain. Metabolite quantification for all simulated and acquired MRS data was performed using LCModel.^45^ The FORTRAN77 codes of LCModel, now freely available as open source, were adapted to provide CRLB floating point values with two decimal digits, instead of the original values provided as integers. All codes (in‐house routines and modified LCModel version) are available upon request. The LCModel basis set for in vivo data was generated using FID‐A.^59^ Basis sets were generated both for each sequence and each TE and included 20 metabolites (Ala, Asc, Asp, Cho, Cr, GABA, Gln, Glu, GPC, GSH, Ins, Lac, NAA, NAAG, PCho, PCr, PE, sIns, Tau, and measured MM baseline). A control node spacing of the spline function for fitting the baseline (dkntmn) of 0.5 was used. SNR was estimated with Matlab by measuring the height of the NAA singlet resonance at 2.0 ppm normalized to the standard deviation of the signal in a peak‐free region (noise region).

### 2.7 Statistical analysis

Metabolite concentrations, CRLB, SNR, and FWHM results are all expressed as mean ± standard deviation. The CoVs (σ/μ) of metabolites concentration across 1000 iterations and of CRLB values across the 50 subgroups were also calculated from simulated datasets. Statistical significance for simulated data (SNRs, FWHMs, CRLBs, CoVs, metabolites concentration ratios) was tested using an unpaired Student's *t* test with equal variance, while significance for in vivo data (SNRs, FWHMs, CRLBs) was tested using a Wilcoxon signed‐rank paired test (GraphPad Software, USA). A threshold of *p* less than 0.05 was considered significant; the following symbols were used to indicate the significance: *p < 0.05, **p < 0.01, ***p < 0.001, and ****p ≤ 0.0001.

## 3 RESULTS

### 3.1 Simulations

#### 3.1.1 Time evolution of selected *J*‐coupled resonances under sLASER and *J*‐sLASER

**FIGURE 2:**
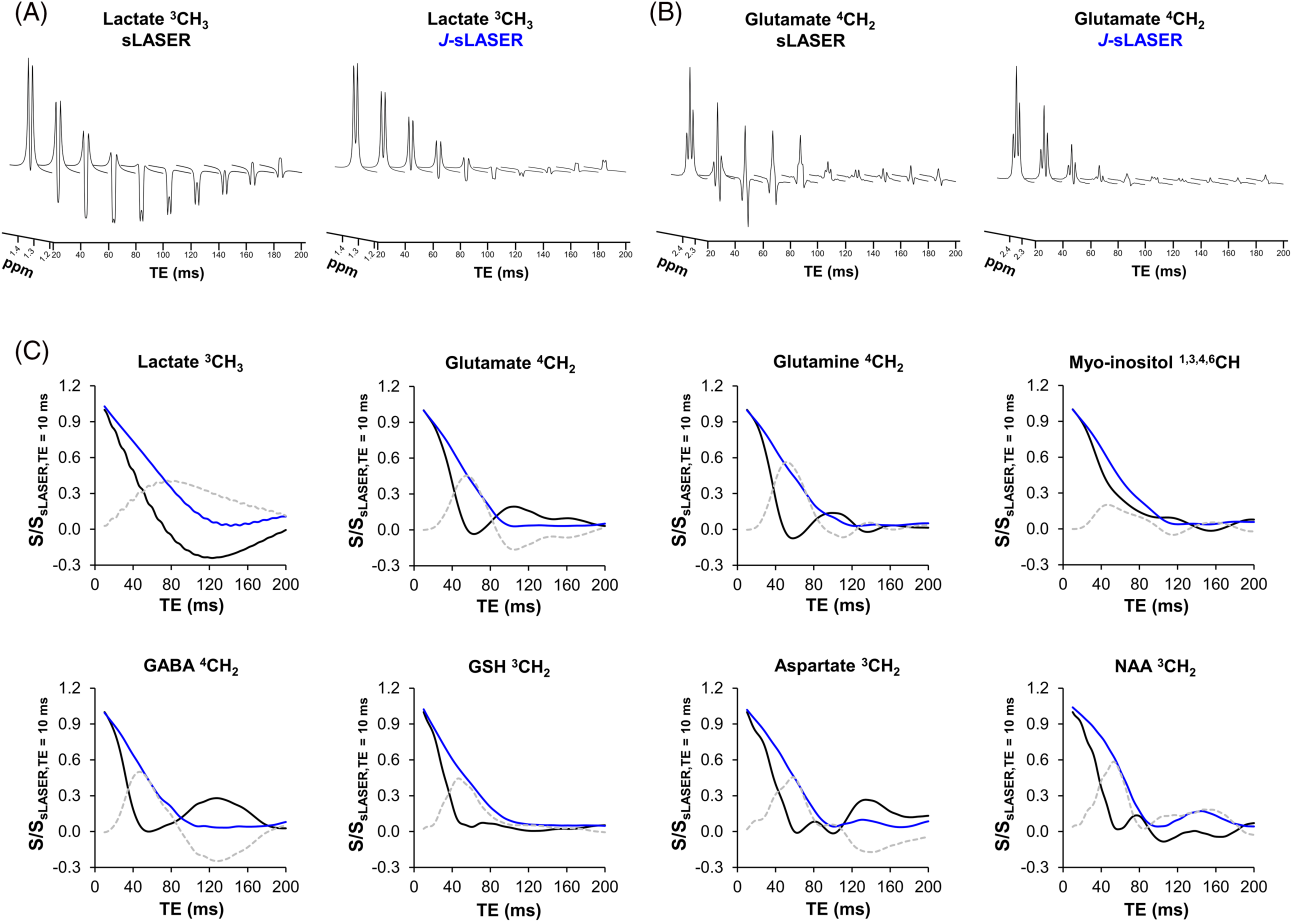
Simulated spectra (S/S) of the (A) ^3^CH_3_ lactate and (B) ^4^CH_2_ glutamate resonances as a function of TE for the sLASER and *J*‐sLASER sequences including T_2_ relaxation; (C) Signal of *J*‐coupled proton groups of eight brain metabolites over a range of TE values (normalized to the signal at TE = 10 ms) as measured for sLASER (black solid line) and *J*‐sLASER (blue line), and the signal difference (*J*‐sLASER – sLASER; light gray dotted line). The superscript number indicated for each metabolite corresponds to the carbon group of the molecule. GABA, gamma‐aminobutyric acid; GSH, glutathione; NAA, N‐acetyl‐aspartate

Figure 2 shows the evolution of the normalized signal of CH, CH_2_, and CH_3_ groups of several brain metabolites under the sLASER and *J*‐sLASER sequences including T2 relaxation. For all groups simulated here, the *J*‐sLASER signal is stronger than the sLASER signal in the range of TE values up to about 80 ms. For all CH and CH_2_ groups the maximum difference between the signal obtained with the two sequences for these resonances is reached at TE values between 50 and 60 ms. The only exception is the Lac CH_3_ resonance, for which the maximum difference occurs at a much longer TE, about 80 ms.

#### 3.1.2 Monte Carlo simulation of spectra acquired with sLASER and *J*‐sLASER

**FIGURE 3:**
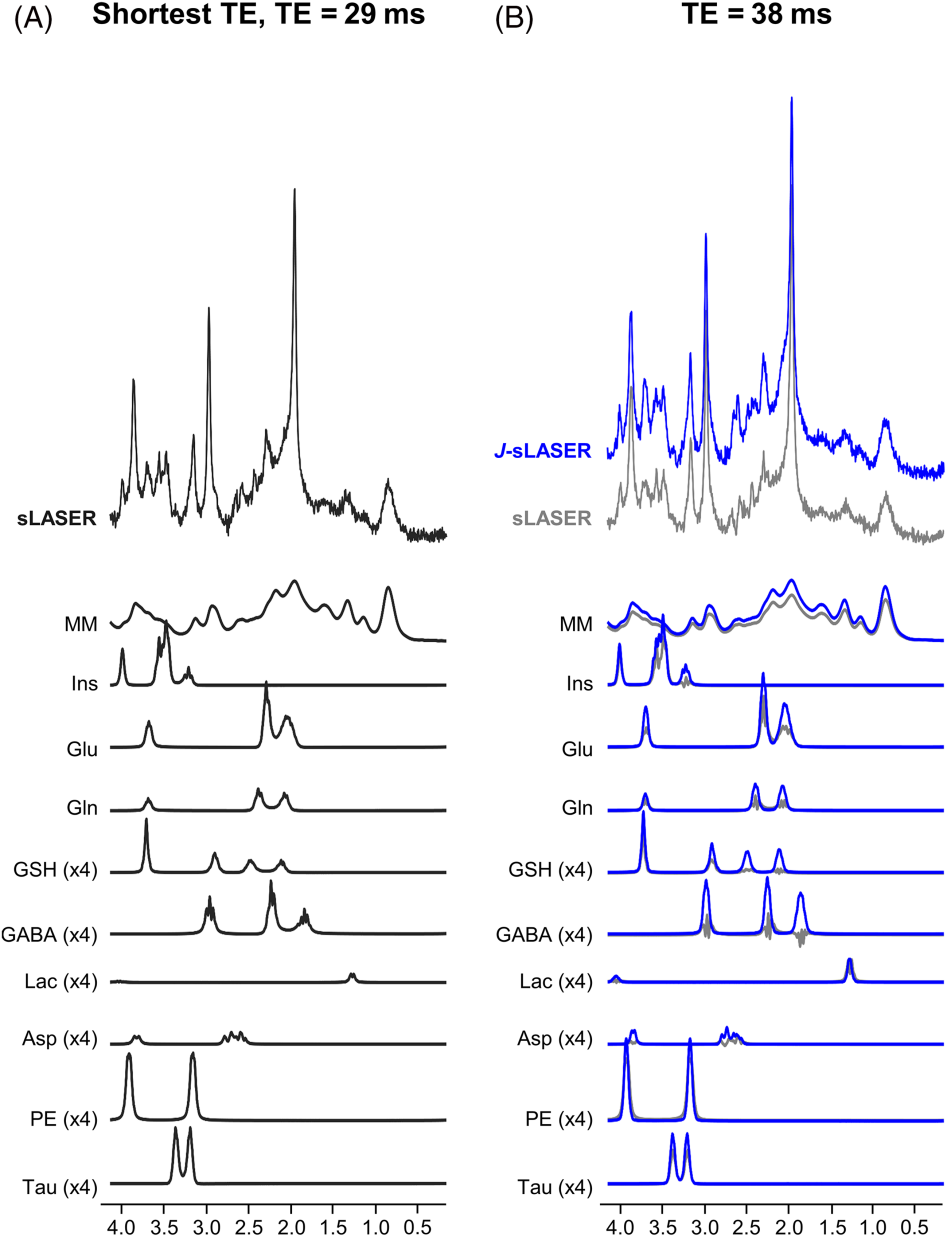
Example of brain‐like simulated spectra with sLASER and *J*‐sLASER sequences used for the Monte Carlo simulations. (A) sLASER spectrum at TE = 29 ms (black); (B) *J*‐sLASER spectrum at TE = 38 ms (blue). The sLASER spectrum at TE = 38 ms is also displayed (gray) to illustrate the increase in signal of *J*‐coupled resonances with *J*‐sLASER at this TE value. Asp, aspartate; GABA, gamma‐aminobutyric acid; Gln, glutamine; Glu, glutamate; GSH, glutathione; Ins, myo‐inositol; Lac, lactate; MM, macromolecule; PE, phosphoethanolamine; Tau, taurine

**FIGURE 4:**
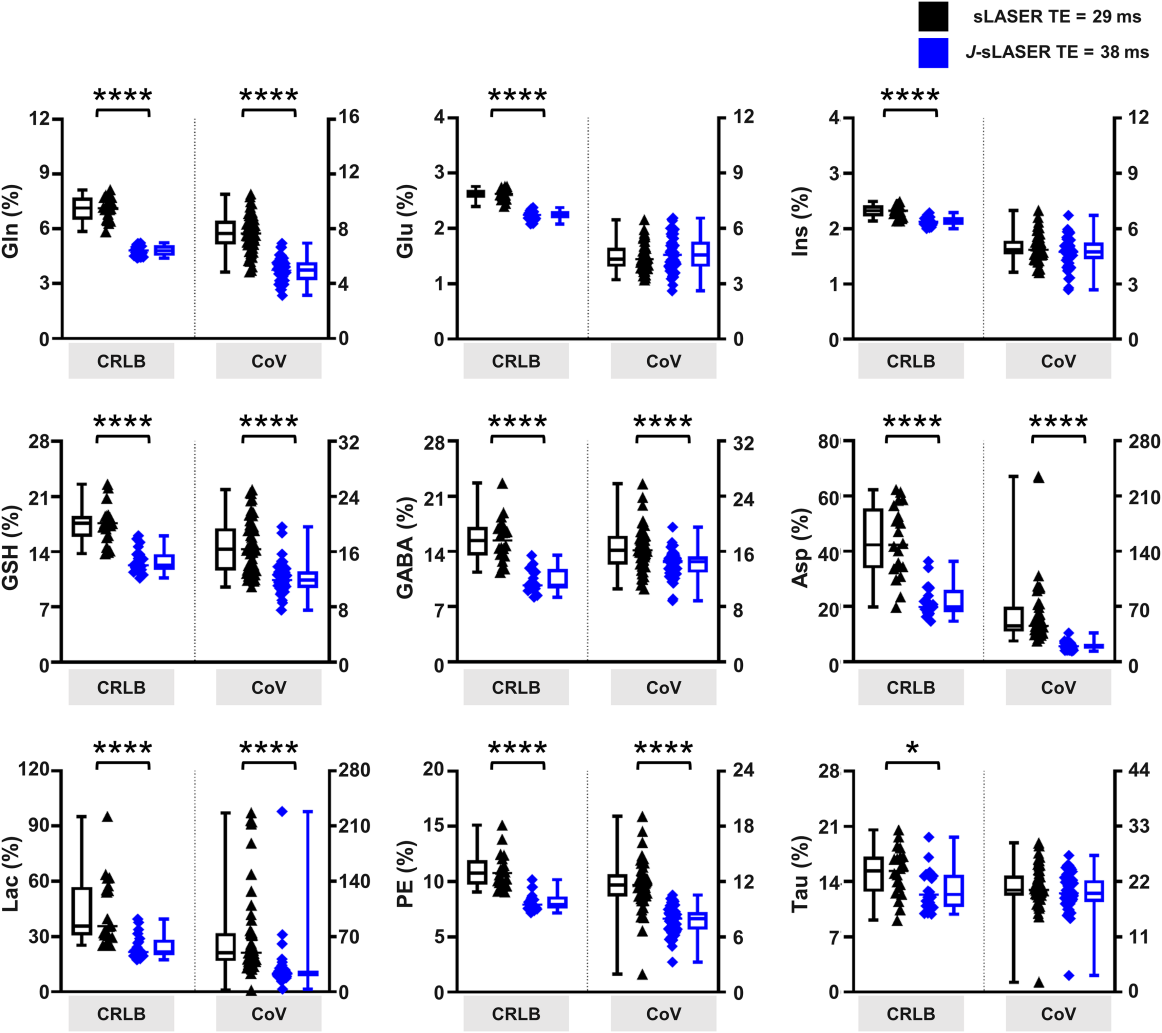
Whisker plots representing the distributions of the LCModel Cramer–Rao lower bound (CRLB) values (%) over 20 repetitions (left) and of the coefficient of variation (CoV) (%) of CRLBs measured over the 20 repetitions for the 50 subgroups (right) for both sLASER at TE = 29 ms (black) and *J*‐sLASER at TE = 38 ms (blue). Statistical significance was evaluated using an unpaired t test with equal variance with *p < 0.05 and ****p ≤ 0.0001. Asp, aspartate; GABA, gamma‐aminobutyric acid; Gln, glutamine; Glu, glutamate; GSH, glutathione; Ins, myo‐inositol; Lac, lactate; PE, phosphoethanolamine; Tau, taurine

Figure 3 shows examples of simulated spectra for the original sLASER sequence at TE = 29 ms, the crusher‐modified sLASER and the *J*‐sLASER sequence at TE = 38 ms. Noise level and line‐broadening on all spectra were adjusted to reach similar SNR (~106) and FWHM (~9 Hz) across sequences. Figure 4 shows whisker plots of the CRLB values obtained from the simulations (20 iterations per sequence). CRLB values for all *J*‐coupled metabolites were significantly lower for the spectra simulated with *J*‐sLASER compared with those with sLASER at the shortest TE. For metabolites such as total NAA (tNAA), tCr, and total Cho (tCho), which are dominated by at least one strong singlet, the CRLB values obtained from both sequences were also significantly lower with the *J*‐sLASER sequence. When comparing output from 1000 repetitions, CRLB values for tNAA and tCho were significantly lower with the *J*‐sLASER sequence (Figure S1). Our 1000 simulations were then divided into 50 subgroups, and we calculated the CoV across the 20 CRLB values obtained for each metabolite across 50 subgroups from the simulated spectra. Figure 4 illustrates differences in CoV between sLASER and *J*‐sLASER datasets. CoVs obtained from the data simulated with the *J*‐sLASER sequence for Gln, GSH, GABA, Asp, and Lac were significantly lower than those obtained from data simulated with sLASER. A full list of the concentrations and CRLBs of metabolites estimated for the different simulation conditions is provided in Tables S1 and S2 and Figure S2.

### 3.2 Phantom experiments

**FIGURE 5:**
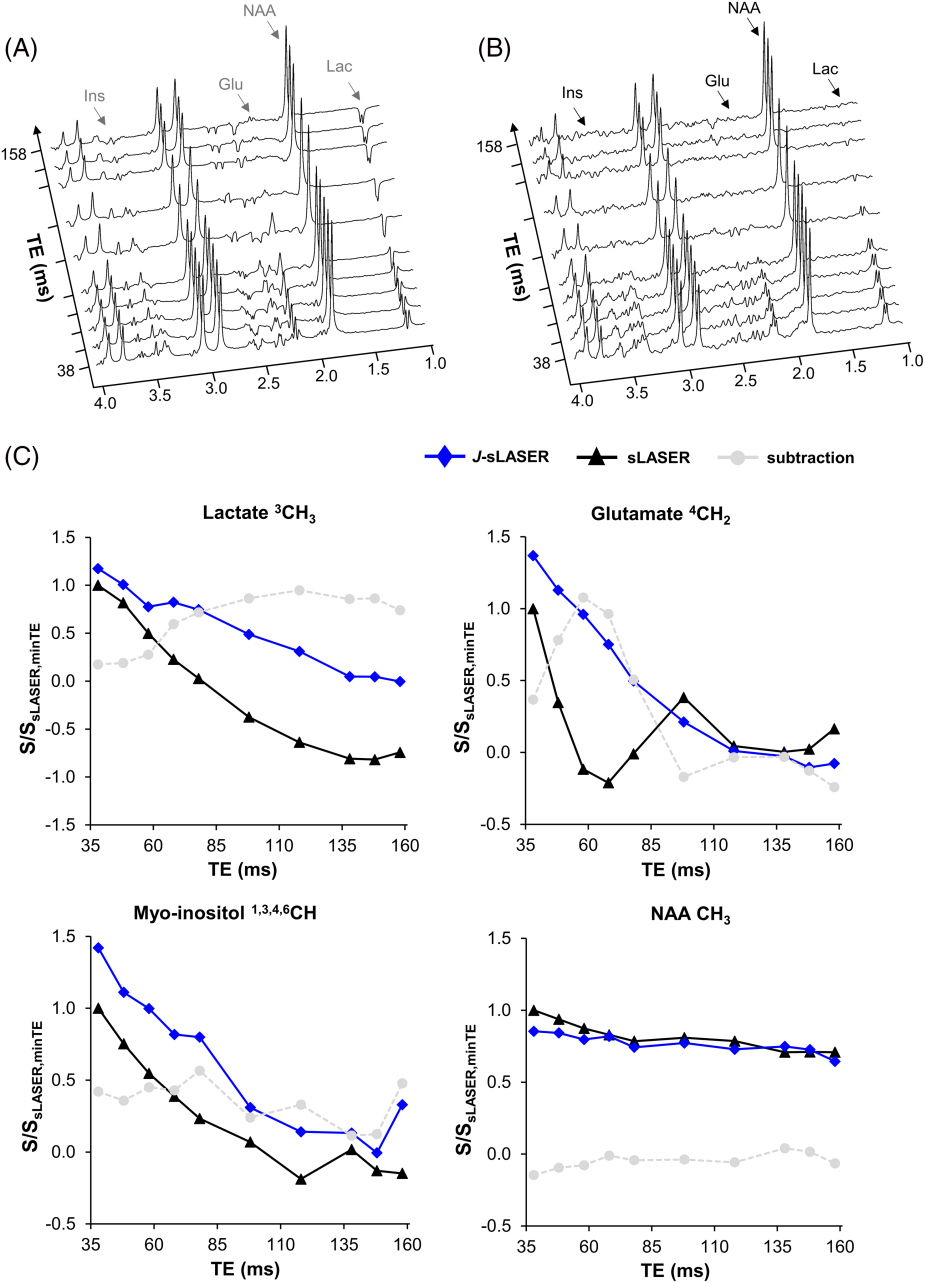
Representative spectra acquired from the BRAINO phantom with the (A) sLASER and (B) *J*‐sLASER sequences with a range of TE values between 38 and 148 ms. The spectra have been zero‐filled and a line‐broadening of 12 Hz has been applied. (C) Quantification of *J*‐coupled and non–*J*‐coupled resonances from data acquired with sLASER (black solid line, triangle) and *J*‐sLASER (blue solid line, diamond). Subtraction of the signal obtained for both sequences is shown in light gray (circle). Glu, glutamate; Ins, myo‐inositol; Lac, lactate; NAA, N‐acetyl‐aspartate

Figure 5 shows spectroscopic data acquired from the phantom with the sLASER and *J*‐sLASER sequences over a range of TE values. The evolution of the *J*‐modulation for the *J*‐coupled metabolites signal closely follows the one obtained with simulation (Figure 2) and shows the same gain in signal with the *J*‐sLASER sequence with TE up to ~80 ms. The signal of singlets, such as the NAA CH_3_ singlet at 2.0 ppm, remained unchanged.

### 3.3 In vivo data: cross‐sectional study

**FIGURE 6:**
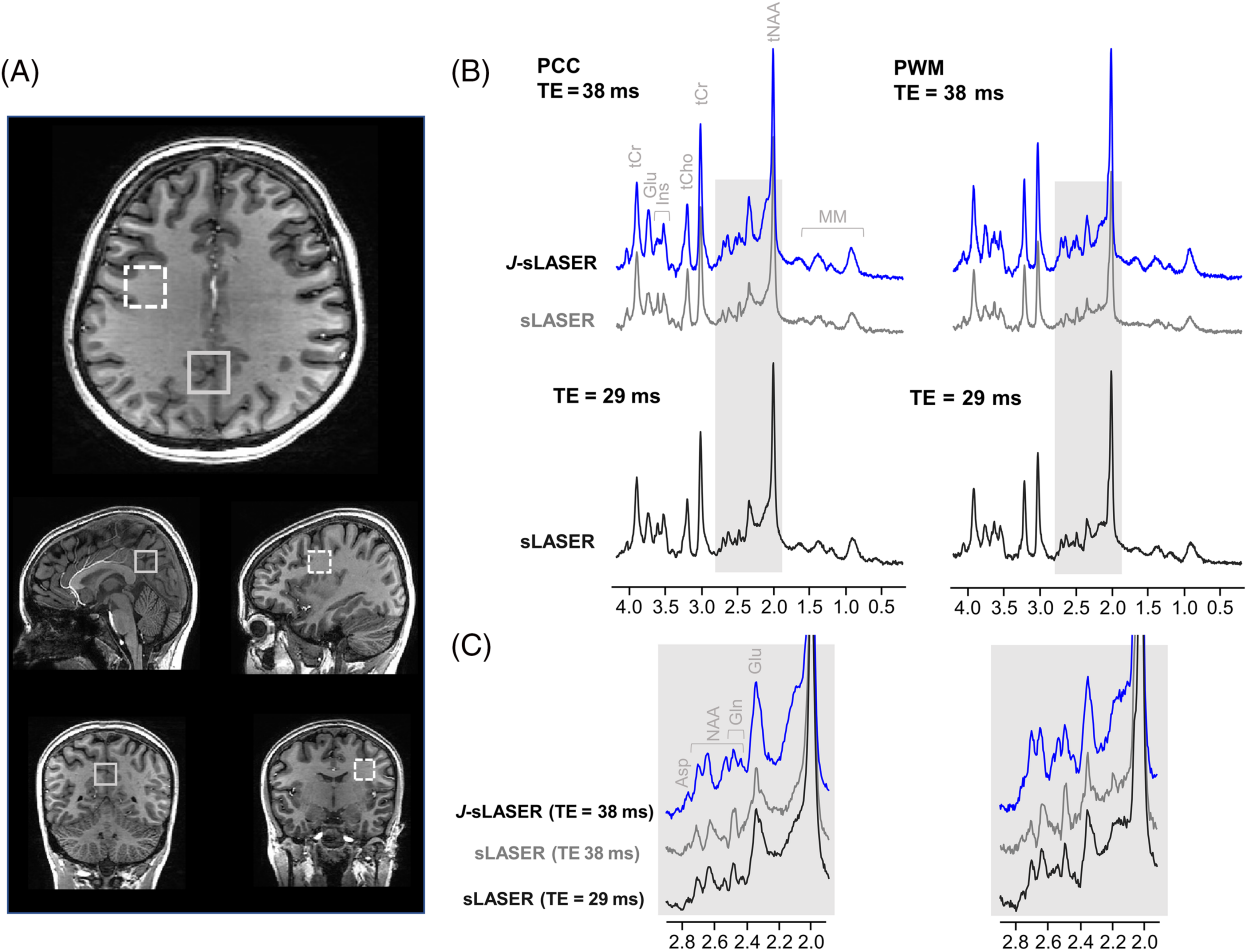
(A) Representative placement of the volume of interest in the PWM region (dashed line) and in the PCC region (solid line). (B) Representative spectra acquired in vivo with the two sequences in both regions. (C) The spectral region between 2.0 and 2.8 ppm shown for sLASER (TE = 29 ms and TE = 38 ms) and *J*‐sLASER (TE = 38 ms), illustrating the signal gain for *J*‐coupled CH_n_ groups. PCC, posterior cingulate cortex; PWM, parietal white matter

Figure 6A shows typical positioning of VOIs in PCC and PWM. The PCC VOI contained 57% ± 5% GM, 36% ± 3% WM, and 7% ± 3% CSF, and the PWM VOI contained 12% ± 3% GM, 85% ± 5% WM, and 3% ± 2% CSF. Representative spectra are shown in Figure 6B,C, illustrating the enhancement of the signal with *J*‐sLASER in both VOIs, particularly for multiplets in the 2.0–2.8 ppm region and the Glu multiplet at 3.8 ppm.

#### 3.3.1 Comparison between sLASER sequences at TE = 29 ms and TE = 38 ms

SNR and FWHM were not statistically different between sLASER at TE = 29 ms and TE = 38 ms (see the full report in Table S3). CRLB values of most metabolites measured with sLASER at TE = 38 ms were slightly increased or remained unchanged compared with those measured with sLASER at the shortest TE = 29 ms (see the full report in Table S3 and Figure S3). The CRLB values of Asp in both PCC and PWM and Lac in PWM were slightly decreased with the sLASER sequence at TE = 38 ms compared with the shortest TE (= 29 ms).

#### 3.3.2 Comparison between sLASER and *J*‐sLASER at the shortest attainable TE

**FIGURE 7:**
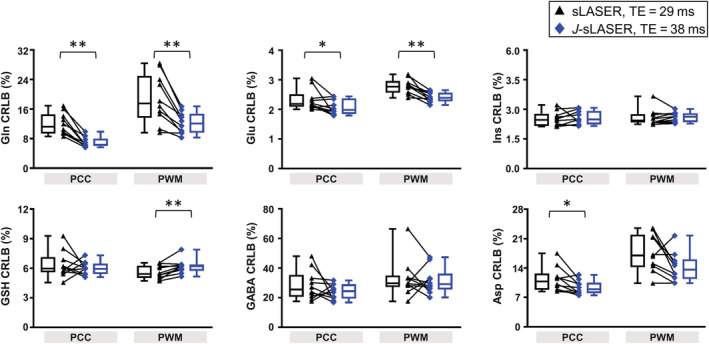
CRLB values estimated for several *J*‐coupled metabolites from sLASER acquisition at shortest TE and *J*‐sLASER acquisitions in both brain regions. Whisker plots (min‐max) and individual data are shown. The black triangle represents the sLASER individual data and the blue diamond represents the *J*‐sLASER individual data. The black line connects data obtained from the same subject. Statistical significance of the differences between sLASER and *J*‐sLASER was evaluated using a Wilcoxon signed‐rank paired test with *p < 0.05 and **p < 0.005. Asp, aspartate; CRLB, Cramer–Rao lower bound; GABA, gamma‐aminobutyric acid; Gln, glutamine; Glu, glutamate; GSH, glutathione; Ins, myo‐inositol; PCC, posterior cingulate cortex; PWM, parietal white matter

SNR was similar with sLASER at TE = 29 ms (228 ± 36 in PCC and 212 ± 27 in PWM) compared with *J*‐sLASER at TE = 38 ms (210 ± 23 in PCC and 221 ± 18 in PWM) in both VOIs, although significantly lower in PCC with *J*‐sLASER. FWHM was similar across sequences and brain regions (10 ± 2/11 ± 2 Hz in PCC and 9 ± 1/9 ± 1 Hz in PWM with sLASER/*J*‐sLASER). See the full report in Table S3. Figure 7 illustrates the analysis of variance in the estimation of several metabolite levels with sLASER at TE = 29 ms and *J*‐sLASER at TE = 38 ms. A significant improvement in metabolite quantification, represented by a significant decrease in CRLB, was observed for Gln and Glu in both VOIs: from 12% ± 3% and 19% ± 7% to 7% ± 1% and 12% ± 3% for Gln and from 2.3% ± 0.4% and 2.8% ± 0.3% to 2.1% ± 0.3% and 2.4% ± 0.2% for Glu in PCC and PWM, respectively. Quantification of Asp significantly improved in the PCC, where the CRLB (Asp) decreased from 11% ± 3% to 9% ± 2%, but not in PWM. However, CRLB (Asp) from the sLASER sequence at TE = 38 ms was also lower when compared with data from sLASER at the shortest TE and *J*‐sLASER in PWM only. Quantification of GSH significantly worsened in the PWM, where the CRLB (GSH) increased from 5.6% ± 0.7% to 6.2% ± 0.7%, but not in PCC. For other *J*‐coupled metabolites such as GSH and GABA in PCC, as well as GABA in PWM, we observed that although the CRLB values obtained with *J*‐sLASER were not significantly lower, the variability in CRLB values across individual measurements was lower for *J*‐sLASER than for sLASER. This becomes evident when examining the CoV of CRLB values for the metabolite estimates obtained with the two sequences. In the PCC, CoV (GSH) was 22% for sLASER and 11% for *J*‐sLASER, CoV (GABA) was 35% and 40% for sLASER in PCC and PWM, respectively, and was 21% and 28% for *J*‐sLASER CoV (GABA) in PCC and PWM, respectively. A full report of the CRLB values and concentrations for all metabolites can be found in Table S3.

### 3.4 In vivo data: repeatability study

**FIGURE 8:**
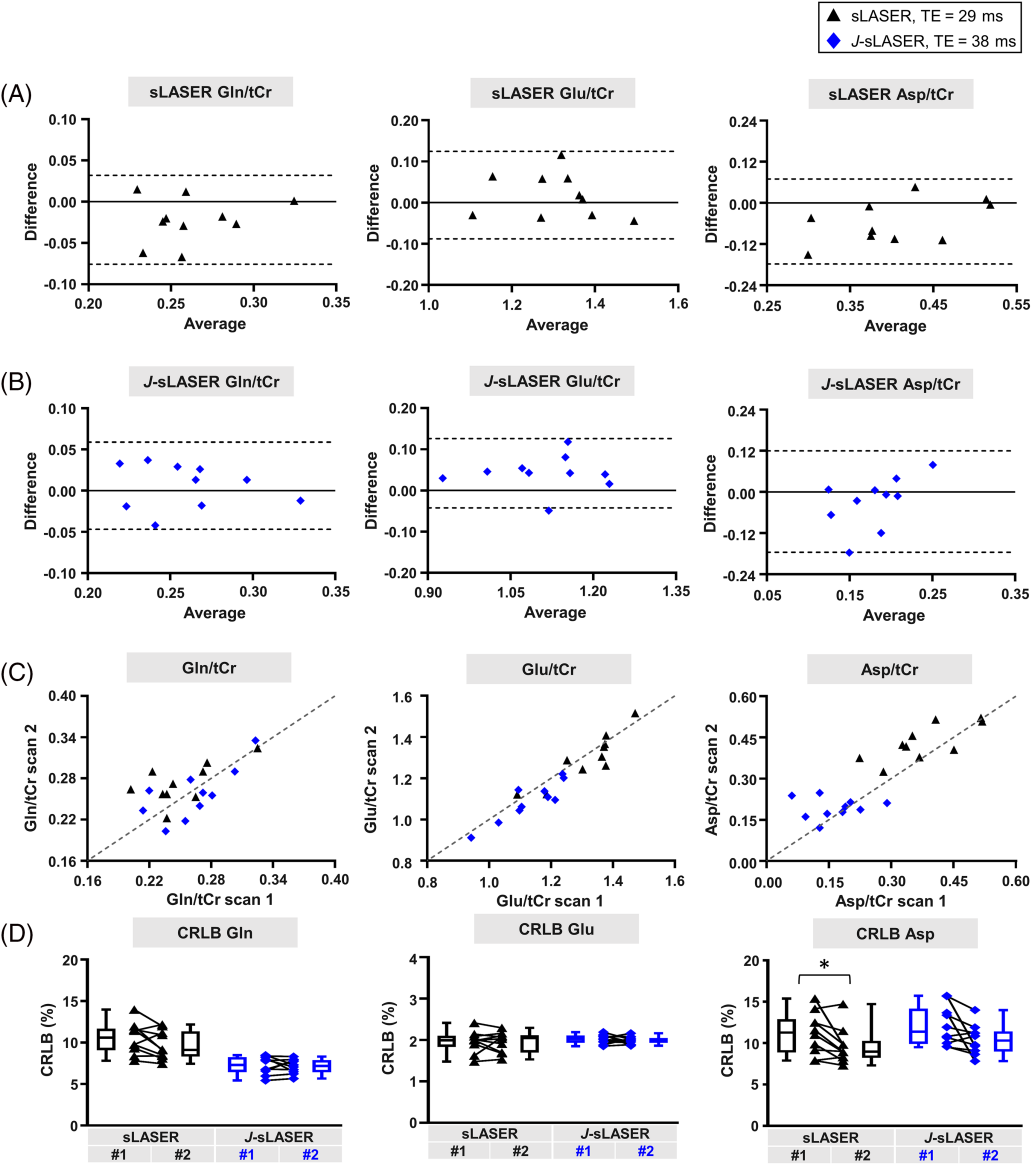
Results of the test–retest analysis for three *J*‐coupled metabolites are shown: Bland–Altman plots for all three metabolites from data acquired with (A) sLASER at shortest TE (black triangles) and (B) *J*‐sLASER (blue diamonds). Dotted lines indicate the 95% limits of agreements. (C) Scatterplots for all three metabolites for both sLASER at shortest TE (black triangles) and *J*‐sLASER (blue diamonds). The dotted line indicates the identity line. (D) Cramer–Rao lower bound (CRLB) values estimated from sLASER acquisition at shortest TE and *J*‐sLASER acquisitions. Whisker plots (min‐max) and individual data are shown. The black triangles represent the sLASER individual data and the blue diamonds represent the *J*‐sLASER individual data. The black line connects data obtained from the same subject. Statistical significance of the differences between sLASER and *J*‐sLASER was evaluated using a Wilcoxon signed‐rank paired test with *p < 0.05. Asp, aspartate; Gln, glutamine; Glu, glutamate; tCr, total creatine

In the first set of acquisitions, the tissue volume fractions within the VOI were GM: 68% ± 5%, WM: 27% ± 4%, and CSF: 5% ± 3%. In the second set of acquisitions, the tissue fractions were GM: 69% ± 5%, WM: 26% ± 5%, and CSF: 5% ± 3%. Within‐subject CoV values for sLASER at the shortest TE and *J*‐sLASER were similar. While the value for Asp was higher with *J*‐sLASER (33.6%) compared with sLASER at shortest TE (16.2%), it was lower for Gln with *J*‐sLASER (7.7%) compared with sLASER (9.7%). A full listing of CRLB values, concentrations, and within‐subject CoVs for all metabolites can be found in Table S4. Bland–Altman and scatterplots, as well as CRLB values, for three *J*‐coupled metabolites, are reported in Figure 8 to illustrate the repeatability of the measurement with both sLASER and *J*‐sLASER. No statistical significance in CRLB values was observed between the two acquisitions for all reported metabolites for both sequences, with the exception of Asp with the sLASER sequence.

## 4 DISCUSSION

We implemented a *J*‐refocused variant of the sLASER sequence (*J‐*sLASER) on a 7 T MRI scanner and showed that in vivo quantification of the *J*‐coupled metabolites Glu, Gln, and Asp significantly improved. The *J*‐sLASER sequence combines the benefits of the sLASER sequence at UHF, in that it reduces the CSDE and refocusing inhomogeneity within the VOI, and is thus better suited for UHF than the *J*‐refocused PRESS variant.^33^

The modifications on the vendor‐supplied sLASER sequence required to implement *J*‐sLASER are simple (Figure 1), but it is important to keep in mind that several steps are required to avoid significant loss in signal and introduction of strong artifacts. First and foremost, it is essential to minimize the contribution of the out‐of‐volume signal generated by the added π/2 RF pulse. In standard sLASER the residual transverse magnetization generated by the π pulses is efficiently suppressed with a combination of spoiler gradients and phase cycling. To avoid loss of signal due to the generation of a stimulated echo, the *J*‐sLASER sequence requires that the chemical shift component of the transverse magnetization be fully refocused at the time of the application of the additional π/2 pulse, thus precluding the addition of spoiler gradients around this pulse. This also prevents using an optimized spoiler gradient scheme for the pulse sequence as a whole^60^ and necessitates a separate spoiler gradient scheme for the first and second pair of π pulses, and by that possibly diminishing the overall efficiency of the spoilers. Thus, proper phase cycling and efficient outer‐volume suppression, especially in the upfield lipid region, are essential for obtaining artifact‐free spectra that match the SNR of standard sLASER with the same TE. The detrimental contribution of out‐of‐volume signal from the lipid region can be further minimized by using a spectral‐spatial π/2 RF pulse matching the spectral region of the *J*‐coupled resonances, rather than the narrow square pulse we used here. Our choice aimed to minimize the added time caused by the addition of the pulse, and we plan on further exploring the optimal choice for the *J*‐refocusing pulse. The requirement of a fully refocused transverse magnetization during the delivery of the π/2 RF pulse also precludes the addition of “perfect echo” π/2 RF pulses during the first and third π ‐ π RF intervals: the application of a single π adiabatic refocusing RF pulse results in a spatially dependent phase dispersion at the expected echo formation time^38^ and will result in a stimulated echo‐type signal loss of 50% for each pulse.

Deviations from the nominal flip angle of the incorporated π/2 RF pulse, due to inaccuracy in flip angle determination or B_1_^+^ inhomogeneity, could result in loss in signal due to suboptimal refocusing. We performed simulations with variations in the flip angle up to 20% from the nominal value by introducing a scaling factor ranging from 0.8 to 1 to the flip angle of the π/2 RF pulse, and evaluated its impact on CRLB estimations. We provide a description of the simulation and results in Figure S6. With the exception of CRLB (Tau) and CRLB (Ins) at the highest deviation from the nominal flip angle (scaling factor = 0.8), the CRLB values obtained for all *J*‐sLASER conditions were significantly lower than when measured using sLASER data and did not significantly change with the flip angle within the range provided by the simulation.

Improvement in metabolite quantification with the *J*‐sLASER sequence compared with the standard sLASER sequence was observed across *J*‐coupled metabolites in the simulation results, as well as in the in vivo, particularly for the quantification of Gln and Glu in both brain regions and that of Asp in the PCC. This significant improvement was achieved despite the 9‐ms difference in TE between *J*‐sLASER and sLASER at minimum TE (38 and 29 ms, respectively), with which both simulations and experiments were performed. Note that for both the sLASER and *J*‐sLASER sequence the minimum TE depends on several additional factors, including angulation of the VOI and maximum B_1_, and thus may vary across protocols. In the in vivo measurements, the difference in TE led to a significant ~8% decrease in the overall SNR in PCC between the two acquisitions (Table S3). Some of the *J*‐coupled metabolites for which the quantification was improved in the simulation, for example, GABA and GSH, did not exhibit similar improvement in vivo. This can be at least partly explained by the added variance in the in vivo dataset due to the difference in concentrations across subjects. It is also possible that the difference in variance in the CRLB values for the two acquisitions is as critical as the difference in the mean CRLB value in determining the improvement provided by *J*‐sLASER. This can be appreciated by looking at the CRLB box plots of the simulation (Figures 4 and S1) and the in vivo data (Figure 7). In both instances it can be seen that for several metabolites the variance in the CRLB values is larger for sLASER than for *J*‐sLASER. This can also be appreciated from the measure of the CoV over CRLB values from simulated datasets in Figure 4, which show a reduced variance for several metabolites, particularly GSH, Asp, Lac, and PE. For other metabolites, the CoV over CRLBs remained unchanged, except for Glu and Ins, for which the CoV increased with both. This observation is consistent with the in vivo data across metabolites. The mean CRLBs were significantly lower with *J*‐sLASER for several *J*‐coupled metabolites, including Gln and Glu in both regions, Asp in the PCC, and GSH in PWM. For other metabolites, such as GSH in the PCC, Asp in PWM, and GABA in both regions, for which no statistically significant difference in mean CRLB was found between the spectra acquired with the two sequences, the variance across CRLB values was reduced with *J*‐sLASER. The larger variance in CRLB values for sLASER may explain why a statistically significant difference in mean CRLB between the two sequences was not reached, and in itself may be an indicator for a more reproducible performance of *J*‐sLASER in the quantification of *J*‐coupled metabolites. When doubling our sampling size for the PCC experiments by combining results from the cross‐sectional study (n = 10) and repeatability study (n = 10), we observed that the difference between CRLB (Gln) obtained from *J*‐sLASER and sLASER was even more significant (Figure S4). While no significant differences in CRLB (Glu) and CRLB (Asp) were observed (compared with the results obtained in the cross‐sectional study alone), data shown in Figure S4 indicate a substantially smaller variance in the CRLBs obtained from the *J*‐sLASER data compared with those obtained from the sLASER. The large variance in CRLB values obtained from the sLASER data not only explains the lack of statistically significant differences between the CRLBs obtained from the two spectroscopic methods, but also serves as an additional incentive for using the *J*‐sLASER sequence.

The simulation of the signal of the *J*‐coupled resonances as a function of TE, as well as the results of the TE‐dependent phantom acquisitions, show that for several of the *J*‐coupled resonances of metabolites the maximum difference in signal between sLASER and *J*‐sLASER occurs between 50 and 60 ms, as shown similarly by others with other acquisition methods.^13^ This suggests a possibility of implementing an editing approach by taking the difference between sLASER and *J*‐sLASER spectra acquired at a TE of ~56 ms. We investigated whether this approach is advantageous using Monte Carlo simulation of brain‐like spectra with sLASER and *J*‐sLASER at TE = 56 ms. As reported in Figure S5, the CRLB values obtained with the simulation of the editing approach were higher than those obtained with simulated data with *J*‐sLASER at the shortest TE across *J*‐coupled metabolites. The only exception was Asp, for which the CRLB was 9.2% ± 1.1% with editing, and 23.8% ± 5.8% with *J*‐sLASER at TE = 38 ms. As the editing approach did not offer significant advantages over the direct acquisition with *J*‐sLASER, we did not further investigate the editing strategy in vivo. As the simulations indicate potential improvement in Asp quantification, it may be of interest to investigate this approach in conditions where Asp is the main target of research.

The benefits offered by the *J*‐sLASER sequence can improve the accuracy and repeatability of measurement of small changes in metabolites in functional MRS (fMRS) experiments. In particular, the improvement in quantification of Gln may in turn improve the independent quantification of Glu, the main excitatory neurotransmitter in the brain, shown to be modulated during functional tasks,^52^, ^61^, ^62^, ^63^, ^64^ and will simultaneously help to track possible modulations of Gln, which is a key player in the glutamatergic cycle in astrocytes.^65^ While quantification of Gln is demonstrably improved with *J*‐sLASER, accurate quantification of other *J*‐coupled neurometabolites such as GABA and GSH remains challenging. Accuracy and precision in the quantification of these metabolites depends on several factors, one of which is the degree of linear dependence in their spectral contributions to Gln, which greatly depends on the sequence used for the acquisition. An indication of this effect can be seen in the reduced variance in CRLB values for both GABA and GSH with *J*‐sLASER compared with sLASER, and in future studies we intend to examine this effect in more depth and evaluate the improvement it may offer in functional studies. Similarly, the apparent improvement in quantification of Asp in GM should be further tested in fMRS settings, both using the *J*‐sLASER at the shortest TE and the edited *J*‐sLASER sequence.^66^ Finally, our simulations suggest that *J*‐sLASER at short TE is expected to improve the detection of Lac, an important target of research in fMRS studies. However, this improvement might be counterbalanced by the partial refocusing of *J*‐coupled resonances in the overlapping MM signal. The overall gains of using *J*‐sLASER for Lac quantification at short TE thus remain to be further investigated.

Although we focused here on UHF, the *J*‐sLASER sequence also offers strong potential at lower clinical magnetic field, such as 3 T MR scanners. At lower clinical magnetic field (3 T), Glu and Gln cannot be separated and are commonly quantified as Glx (Glu + Gln). The *J*‐sLASER sequence might offer a potential alternative to better quantify Glu and Gln separately at 3 T. Further investigations are needed to study the potential of this sequence when measuring metabolic changes.

## 5 CONCLUSION

We illustrated the improvement in detection of *J*‐coupled resonances in the human brain at UHF with the *J*‐sLASER sequence. We demonstrated a straightforward implementation of the *J*‐sLASER sequence in single volume mode on our 7 T MRI scanner, and obtained significant improvement in the quantification of Glu, Gln, and Asp in the human brain. The *J*‐sLASER sequence offers benefits that can be used to improve the measurement of small metabolic changes in *J*‐coupled metabolites in experiments such as fMRS, and can be easily incorporated in spectroscopic imaging sequences, similar to the incorporation of *J*‐refocused PRESS in such sequences. The positioning of spoiler gradients as well as proper phase cycling are crucial to prevent strong out‐of‐volume signal from lipids and water generated by added π/2 RF pulse.

## CONFLICTS OF INTEREST

The authors declare no conflicts of interest.

## Supporting information


**Table S1:** Mean ± SD for concentration (ratio to tCr) and CRLB values estimated after fitting 20 brain‐like simulated spectra for sLASER and *J*‐sLASER sequence at TE = 29 ms and 38 ms. Results suggest that *J*‐sLASER at minimal TE provides the most accurate quantification for *J*‐coupled metabolites without affecting non *J*‐coupled metabolites.
**Table S2**: Mean ± SD for concentration (ratio to tCr) and CRLB values estimated after fitting 1,000 brain‐like simulated spectra for sLASER and *J*‐sLASER sequence at TE = 29 ms and 38 ms. Results suggest that *J*‐sLASER at minimal TE provides the most accurate quantification for *J*‐coupled metabolites without affecting non *J*‐coupled metabolites.
**Table S3:** Cross‐sectional study. SNR, FWHM, concentration normalized to tCr and CRLB values (mean±s.d.) in both VOIs and three acquisitions are reported.
**Table S4:** Repeatability study. SNR, FWHM, concentration normalized to tCr and CRLB values (mean±s.d.) for both acquisitions performed with sLASER at shortest TE and *J*‐sLASER are reported. The within‐subject coefficient of variation for metabolite level (CoV) is calculated as in de Boer et al, J. Magn. Reson. Imaging 2021
**Figure S1**: Violin plots representing the distributions of the LCModel CRLB values (%) over 1,000 repetitions for both the sLASER at TE = 29 ms (black plots) and *J*‐sLASER at TE = 38 ms (blue plots). The black triangles and blue diamonds represent the CoV values (%) calculated from the LCModel concentration values. CRLB values above 100 were discarded here, resulting in removal of 43 and 51 data points for Asp and Lac respectively for the sLASER sequence at TE = 29 ms and 2 data points for Lac for the *J*‐sLASER sequence. No data points were removed for the *J*‐sLASER sequence.
**Figure S2:** (A) Concentrations normalized to the concentration of tCr and corrected for T2 relaxation for the shortest TE sLASER (black) and *J*‐sLASER (blue) at TE = 38 ms sequences. (B) Correlation between concentration ratios of each metabolites (one color per metabolite) for both sequences, showing almost a 1:1 relation. Statistical significance between both sequences (represented with * were evaluated using a unpaired *t*‐ test *p < 0.05, **p < 0.005, ***p < 0.001, ****p ≤ 0.0001.
**Figure S3:** CRLB values estimated for several *J*‐coupled metabolites from sLASER acquisition at shortest TE and TE = 38 ms in both regions are represented. Whisker plots (min‐max) and individual data are shown. The black triangle represents the sLASER at TE = 29 ms individual data and the gray diamond represents the sLASER at TE = 38 ms individual data. The black line connect data from same individual. Statistical significance between both sequences (represented with *) were evaluated using a Wilcoxon signed‐rank paired test with *p < 0.05 and **p < 0.005.
**Figure S4:** Data acquired with sLASER and *J*‐sLASER sequences in PCC from the cross‐sectional and repeatability studies were combined. CRLB values estimated for several *J*‐coupled metabolites are displayed. Whisker plots (min‐max) and individual data are shown. The black triangle represents the sLASER individual data and the blue diamond represents the *J*‐sLASER individual data. The black line connects data from same individual. Statistical significance between sLASER and *J*‐sLASER (represented with *) were evaluated using a Wilcoxon signed‐rank paired test with *p < 0.05 and **p < 0.005.
**Figure S5:** (A) Representative ‘brain‐like’ spectra simulated for sLASER, *J*‐sLASER and edited (subtraction of *J*‐sLASER and sLASER) at TE = 56 ms. (B) Whisker plots (5–95 percentile) representing the distributions of the LCModel CRLB values (%) over 1,000 repetitions for both the *J*‐sLASER at TE = 38 ms and edited‐*J*‐sLASER at TE = 56 ms.
**Figure S6:** We explored the effect of deviations from the nominal flip angle of the π/2 square RF pulse applied in the *J*‐sLASER sequence (due to B1^+^ inhomogeneities). We simulated 100 ‘brain‐like’ spectra for the *J*‐sLASER sequence at TE = 38 ms with 5 scaling factors for the π/2 square RF pulse: 0.8, 0.85, 0.9, 0.95, and 1 (resulting in the following flip angles: 72°, 76.5°, 81°, 85.5°, and 90°). We compared the CRLBs measured under these conditions to the CRLBs estimated from 100 ‘brain‐like’ spectra generated with the sLASER sequence at TE = 29 ms. Whisker plots (min‐max) represent the distributions of the LCModel CRLB values (%) over 100 repetitions. CRLB values above 100 were discarded here, resulting in removal of 5 data points for Asp for the sLASER sequence at TE = 29 ms. An unpaired Students *t* test with equal variance (GraphPad Software, USA) was used to compare each *J*‐sLASER acquisition to the sLASER acquisition. A threshold of p < 0.05 was considered significant, the following symbols where used to indicate the significance: *p < 0.05, **p < 0.01, ***p < 0.001, ****p ≤ 0.0001. Except CRLB (Tau) and CRLB (Ins) at the lowest scaling factor (0.8), CRLB values measured for all *J*‐sLASER conditions were significantly lower than when measured using sLASER data.
