# Supplementary material for: Improved detection limits of J‐coupled neurometabolites in the human brain at 7 T with a J‐refocused sLASER sequence
Source: NMR Biomed. 2022 Aug 10;35(12):e4801. doi: 10.1002/nbm.4801 (PMC9788253; doi:10.1002/nbm.4801)
Supplement: Supplementary file 1 — Table S1: Mean ± SD for concentration (ratio to tCr) and CRLB values estimated after fitting 20 brain‐like simulated spectra for sLASER and J‐sLASER sequence at TE = 29 ms and 38 ms. Results suggest that J‐sLASER at minimal TE provides the most accurate quantification for J‐coupled metabolites without affecting non J‐coupled metabolites. Table S2: Mean ± SD for concentration (ratio to tCr) and CRLB values estimated after fitting 1,000 brain‐like simulated spectra for sLASER and J‐sLASER sequence at TE = 29 ms and 38 ms. Results suggest that J‐sLASER at minimal TE provides the most accurate quantification for J‐coupled metabolites without affecting non J‐coupled metabolites. Table S3: Cross‐sectional study. SNR, FWHM, concentration normalized to tCr and CRLB values (mean±s.d.) in both VOIs and three acquisitions are reported. Table S4: Repeatability study. SNR, FWHM, concentration normalized to tCr and CRLB values (mean±s.d.) for both acquisitions performed with sLASER at shortest TE and J‐sLASER are reported. The within‐subject coefficient of variation for metabolite level (CoV) is calculated as in de Boer et al, J. Magn. Reson. Imaging 2021 Figure S1: Violin plots representing the distributions of the LCModel CRLB values (%) over 1,000 repetitions for both the sLASER at TE = 29 ms (black plots) and J‐sLASER at TE = 38 ms (blue plots). The black triangles and blue diamonds represent the CoV values (%) calculated from the LCModel concentration values. CRLB values above 100 were discarded here, resulting in removal of 43 and 51 data points for Asp and Lac respectively for the sLASER sequence at TE = 29 ms and 2 data points for Lac for the J‐sLASER sequence. No data points were removed for the J‐sLASER sequence. Figure S2: (A) Concentrations normalized to the concentration of tCr and corrected for T2 relaxation for the shortest TE sLASER (black) and J‐sLASER (blue) at TE = 38 ms sequences. (B) Correlation between concentration ratios of each metabolites (one color per [file NBM-35-e4801-s001.docx]

**Supplementary materials**

***Table S1****: Mean ± SD for concentration (ratio to tCr) and CRLB values estimated after fitting 20 brain-like simulated spectra for sLASER and J-sLASER sequence at TE=29ms and 38ms. Results suggest that J-sLASER at minimal TE provides the most accurate quantification for J-coupled metabolites without affecting non J-coupled metabolites.*

|  | sLASER TE=29 ms | | sLASER TE=38 ms | | *J*-sLASER TE=38 ms | |
| --- | --- | --- | --- | --- | --- | --- |
| SNR  FWHM | 104±5  9.4±0.6 Hz | | 107±6  9.2±0.5 Hz | | 109±5  9.2±0.5 Hz | |
|  | **/Cr** | **CRLB (%)** | **/Cr** | **CRLB (%)** | **/Cr** | **CRLB (%)** |
| Ins | 0.709±0.014 | 2.32±0.11 | 0.681±0.012 | 2.77±0.09 | 0.726±0.011 | 2.14±0.08 |
| Glu | 1.068±0.024 | 2.62±0.10 | 1.078±0.027 | 3.43±0.09 | **1.068±0.019** | **2.24±0.09** |
| Gln | 0.427±0.031 | 7.09±0.62 | 0.403±0.053 | 14.63±1.62 | **0.388±0.020** | **4.81±0.29** |
| GSH | 0.084±0.010 | 17.48±2.45 | 0.076±0.011 | 21.37±3.20 | **0.103±0.011** | **12.81±1.54** |
| GABA | 0.232±0.036 | 15.49±2.77 | 0.179±0.040 | 28.49±6.60 | **0.190±0.022** | **10.34±1.47** |
| Lac | 0.043±0.015 | 44.01±18.24 | 0.041±0.018 | 62.72±47.72 | **0.045±0.010** | **24.54±6.56** |
| Asp | 0.116±0.037 | 43.27±12.85 | 0.072±0.033 | 138.07±205.83 | **0.134±0.030** | **21.87±5.74** |
| PE | 0.299±0.037 | 10.95±1.59 | 0.298±0.038 | 11.87±1.45 | **0.234±0.023** | **8.17±0.82** |
| Tau | 0.121±0.018 | 15.16±3.07 | 0.111±0.011 | 18.03±2.65 | **0.128±0.016** | **13.00±2.62** |
| tNAA | 1.435±0.024 | 1.06±0.05 | 1.397±0.026 | 0.96±0.01 | 1.466±0.022 | 1.00±0.04 |
| tCr | - | 1.49±0.08 | - | 1.33±0.04 | - | 1.43±0.05 |
| tCho | 0.088±0.004 | 5.21±0.45 | 0.088±0.003 | 4.72±0.45 | 0.092±0.005 | 4.72±0.37 |

***Table S2****: Mean ± SD for concentration (ratio to tCr) and CRLB values estimated after fitting 1000 brain-like simulated spectra for sLASER and J-sLASER sequence at TE=29ms and 38ms. Results suggest that J-sLASER at minimal TE provides the most accurate quantification for J-coupled metabolites without affecting non J-coupled metabolites.*

|  | sLASER TE=29 ms | | sLASER TE=38 ms | | *J*-sLASER TE=38 ms | |
| --- | --- | --- | --- | --- | --- | --- |
| SNR  FWHM | 104±5  9.2±0.5 Hz | | 108±5  9.1±0.4 Hz | | 108±6  9.2±0.5 Hz | |
|  | **/Cr** | **CRLB (%)** | **/Cr** | **CRLB (%)** | **/Cr** | **CRLB (%)** |
| Ins | 0.710±0.013 | 2.26±0.13 | 0.676±0.015 | 2.80±0.14 | 0.729±0.014 | 2.11±0.12 |
| Glu | 1.061±0.023 | 2.60±0.14 | 1.068±0.031 | 3.48±0.15 | **1.054±0.019** | **2.23±0.13** |
| Gln | 0.431±0.026 | 6.88±0.58 | 0.408±0.051 | 14.55±1.72 | **0.390±0.017** | **4.75±0.29** |
| GSH | 0.083±0.013 | 17.44±3.06 | 0.076±0.014 | 21.79±4.99 | **0.101±0.012** | **13.07±1.66** |
| GABA | 0.241±0.033 | 14.52±2.45 | 0.155±0.035 | 33.45±8.87 | **0.198±0.023** | **9.80±1.43** |
| Lac | 0.041±0.016 | 53.88±81.50 | 0.040±0.018 | 85.00±150.11 | **0.044±0.010** | **25.67±18.37** |
| Asp | 0.112±0.039 | 49.50±52.10 | 0.066±0.042 | 200.88±279.17 | **0.140±0.025** | **20.50±4.27** |
| PE | 0.288±0.030 | 11.14±1.38 | 0.290±0.034 | 12.34±1.55 | **0.230±0.019** | **8.26±0.70** |
| Tau | 0.127±0.017 | 14.03±3.07 | 0.109±0.017 | 18.08±4.23 | **0.133±0.015** | **12.42±2.59** |
| tNAA | 1.430±0.022 | 1.04±0.05 | 1.400±0.022 | 0.97±0.03 | 1.467±0.025 | 0.99±0.06 |
| tCr | - | 1.43±0.08 | - | 1.33±0.06 | - | 1.42±0.08 |
| tCho | 0.089±0.004 | 5.09±0.47 | 0.088±0.04 | 4.76±0.46 | 0.093±0.004 | 4.69±0.42 |

***Table S3:*** *Cross-sectional study. SNR, FWHM, concentration normalized to tCr and CRLB values (mean±s.d.) in both VOIs and three acquisitions are reported.*

|  |  | | PCC | | | PWM | | |
| --- | --- | --- | --- | --- | --- | --- | --- | --- |
|  |  | | sLASER  TE=29ms | sLASER  TE=38ms | *J*-sLASER  TE=38ms | sLASER  TE=29ms | sLASER  TE=38ms | *J*-sLASER  TE=38ms |
| SNR | | 229±36 | | 200±49 | 210±23 | 212±27 | 222±51 | 221±18 |
| FWHM | | 10.4±2.2 | | 10.5±2.1 | 10.5±2.1 | 9.1±1.0 | 9.3±1.5 | 9.3±0.9 |
| Ins | /tCr (-) | 0.72±0.09 | | 0.65±0.06 | 0.62±0.05 | 0.69±0.06 | 0.60±0.05 | 0.61±0.05 |
|  | CRLB (%) | 2.5±0.3 | | 3.0±0.5 | 2.6±0.3 | 2.6±0.4 | 3.0±0.3 | 2.6±0.02 |
| Glu | /tCr (-) | 1.28±0.08 | | 1.09±0.09 | 1.06±0.07 | 1.00±0.10 | 0.88±0.08 | 0.86±0.07 |
|  | CRLB (%) | 2.3±0.4 | | 3.1±0.5 | 2.1±0.3 | 2.8±0.3 | 3.6±0.4 | 2.4±0.2 |
| Gln | /tCr (-) | 0.27±0.07 | | 0.37±0.06 | 0.28±0.03 | 0.17±0.07 | 0.27±0.05 | 0.16±0.04 |
|  | CRLB (%) | 12.0±3.0 | | 17.8±4.1 | 7.0±1.4 | 18.8±6.7 | 20.9±3.6 | 12.2±2.9 |
| GSH | /tCr (-) | 0.21±0.03 | | 0.23±0.02 | 0.19±0.02 | 0.22±0.02 | 0.20±0.02 | 0.18±0.02 |
|  | CRLB (%) | 6.4±1.4 | | 6.5±0.9 | 6.0±0.6 | 5.6±0.7 | 6.4±0.8 | 6.2±0.7 |
| GABA | /tCr (-) | 0.14±0.04 | | 0.16±0.06 | 0.08±0.02 | 0.12±0.03 | 0.10±0.06 | 0.06±0.01 |
|  | CRLB (%) | 28.5±10.1 | | 48.7±58.3 | 24.0±5.1 | 33.0±13.1 | 145.4±300.9 | 31.4±8.9 |
| Lac | /tCr (-) | 0.08±0.03 | | 0.11±0.05 | 0.05±0.02 | 0.08±0.01 | 0.12±0.03 | 0.06±0.01 |
|  | CRLB (%) | 20.4±6.6 | | 19.1±11.3 | 23.0±8.3 | 18.1±2.6 | 14.5±2.9 | 19.6±3.5 |
| Asp | /tCr (-) | 0.43±0.06 | | 0.58±0.06 | 0.35±0.07 | 0.28±0.06 | 0.46±0.06 | 0.24±0.05 |
|  | CRLB (%) | 11.3±2.9 | | 10.4±2.2 | 9.3±1.6 | 17.7±4.5 | 12.3±2.0 | 14.2±3.5 |
| Tau | /tCr (-) | 0.16±0.05 | | 0.14±0.03 | 0.15±0.05 | 0.09±0.04 | 0.06±0.03 | 0.11±0.04 |
|  | CRLB (%) | 11.6±4.6 | | 12.0±4.4 | 11.1±3.4 | 20.2±8.4 | 44.5±44.4 | 15.5±5.1 |
| tNAA | /tCr (-) | 1.28±0.09 | | 1.45±0.12 | 1.33±0.11 | 1.63±0.17 | 1.85±0.20 | 1.69±0.16 |
|  | CRLB (%) | 1.2±0.2 | | 1.0±0.2 | 1.2±0.2 | 1.0±0.1 | 0.8±0.1 | 1.00±0.1 |
| tCr | /tCr (-) | - | | - | - | - | - | - |
|  | CRLB (%) | 1.5±0.3 | | 1.3±0.2 | 1.5±0.2 | 1.4±0.1 | 1.2±0.1 | 1.5±0.1 |
| tCho | /tCr (-) | 0.20±0.07 | | 0.21±0.08 | 0.21±0.08 | 0.30±0.08 | 0.31±0.08 | 0.29±0.08 |
|  | CRLB (%) | 2.4±0.5 | | 2.2±0.4 | 2.3±0.3 | 1.7±0.2 | 1.5±0.2 | 1.7±0.2 |

***Table S4:*** *Repeatability study. SNR, FWHM, concentration normalized to tCr and CRLB values (mean±s.d.) for both acquisitions performed with sLASER at shortest TE and J-sLASER are reported. The within‐subject coefficient of variation for metabolite level (CoV) is calculated as in de Boer et al., J. Magn. Reson. Imaging 2021*

|  |  | |  | |  | | |  |  |  |
| --- | --- | --- | --- | --- | --- | --- | --- | --- | --- | --- |
|  |  | | sLASER  TE=29ms | | | | *J*-sLASER  TE=38ms | | | |
|  |  | | Acq. #1 | Acq. #2 | | CoV (%) | Acq. #1 | | Acq. #2 | CoV (%) |
| SNR | | 237±42 | | 231±37 | |  | 220±30 | | 220±31 |  |
| FWHM | | 10.6±2.2 | | 10.7±2.1 | |  | 9.8±2.0 | | 10.6±2.2 |  |
| Ins | /tCr (-) | 0.72±0.08 | | 0.71±0.10 | | **2.2** | 0.69±0.07 | | 0.69±0.06 | **3.3** |
|  | CRLB (%) | 2.2±0.3 | | 2.2±0.4 | |  | 2.7±0.3 | | 2.6±0.03 |  |
| Glu | /tCr (-) | 1.32±0.11 | | 1.30±0.12 | | **3.0** | 1.13±0.10 | | 1.09±0.09 | **3.7** |
|  | CRLB (%) | 2.0±0.3 | | 2.0±0.2 | |  | 2.0±0.1 | | 2.0±0.1 |  |
| Gln | /tCr (-) | 0.25±0.03 | | 0.27±0.03 | | **9.7** | 0.26±0.03 | | 0.26±0.04 | **7.7** |
|  | CRLB (%) | 10.5±1.9 | | 9.7±1.7 | |  | 7.2±1.1 | | 7.1±0.9 |  |
| GSH | /tCr (-) | 0.19±0.03 | | 0.20±0.03 | | **9.01** | 0.17±0.02 | | 0.18±0.03 | **8.2** |
|  | CRLB (%) | 5.9±1.0 | | 5.8±1.2 | |  | 6.9±0.6 | | 6.3±0.9 |  |
| GABA | /tCr (-) | 0.16±0.04 | | 0.15±0.05 | | **17.9** | 0.12±0.04 | | 0.11±0.03 | **18.2** |
|  | CRLB (%) | 20.9±5.6 | | 23.1±8.1 | |  | 20.6±5.3 | | 21.6±3.1 |  |
| Lac | /tCr (-) | 0.08±0.02 | | 0.07±0.02 | | **20.4** | 0.08±0.03 | | 0.07±0.02 | **31.9** |
|  | CRLB (%) | 17.0±5.5 | | 18.9±8.6 | |  | 22.9±11.6 | | 20.2±5.3 |  |
| Asp | /tCr (-) | 0.38±0.10 | | 0.43±0.07 | | **16.2** | 0.16±0.07 | | 0.19±0.04 | **33.6** |
|  | CRLB (%) | 11.2±2.5 | | 9.6±2.1 | |  | 12.1±2.4 | | 10.4±1.8 |  |
| Tau | /tCr (-) | 0.17±0.05 | | 0.14±0.03 | | **22.2** | 0.22±0.05 | | 0.19±0.05 | **18.7** |
|  | CRLB (%) | 10.2±6.2 | | 10.8±2.8 | |  | 10.6±4.5 | | 14.0±8.9 |  |
| tNAA | /tCr (-) | 1.32±0.06 | | 1.31±0.07 | | **2.4** | 1.47±0.08 | | 1.43±0.07 | **3.0** |
|  | CRLB (%) | 1.0±0.1 | | 1.0±0.2 | |  | 1.2±0.1 | | 1.2±0.1 |  |
| tCr | /tCr (-) | - | | - | | **-** | - | | - | **-** |
|  | CRLB (%) | 1.2±0.1 | | 1.3±0.2 | |  | 1.5±0.3 | | 1.4±0.1 |  |
| tCho | /tCr (-) | 0.18±0.02 | | 0.18±0.01 | | **3.4** | 0.19±0.01 | | 0.19±0.01 | **3.1** |
|  | CRLB (%) | 2.2±0.3 | | 2.1±0.3 | |  | 2.5±0.3 | | 2.4±0.2 |  |

*
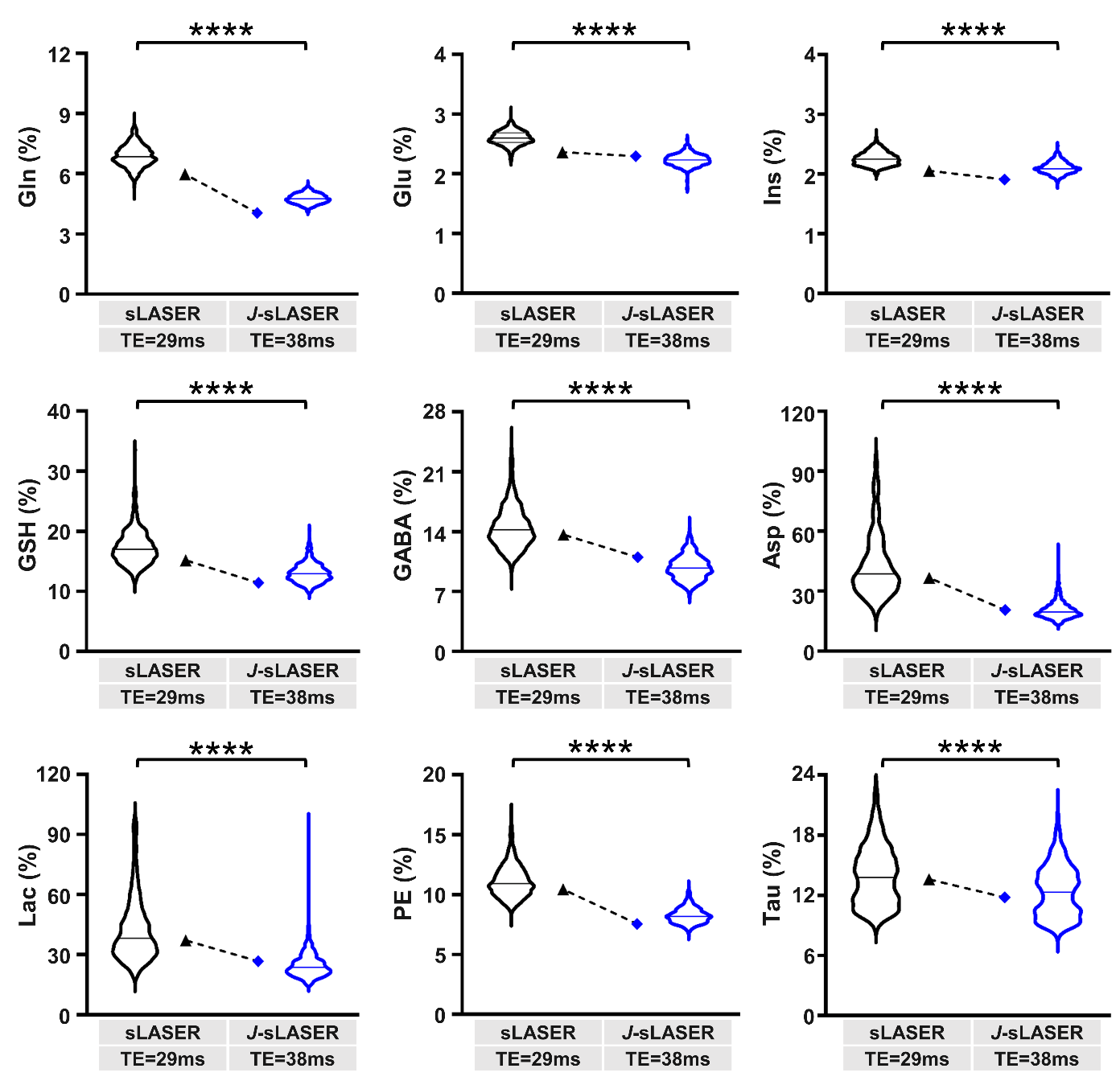
****Figure S1****: Violin plots representing the distributions of the LCModel CRLB values (%) over 1000 repetitions for both the sLASER at TE=29ms (black plots) and J-sLASER at TE=38ms (blue plots). The black triangles and blue diamonds represent the CoV values (%) calculated from the LCModel concentration values. CRLB values above 100 were discarded here, resulting in removal of 43 and 51 data points for Asp and Lac respectively for the sLASER sequence at TE=29ms and 2 data points for Lac for the J-sLASER sequence. No data points were removed for the J-sLASER sequence.*

***
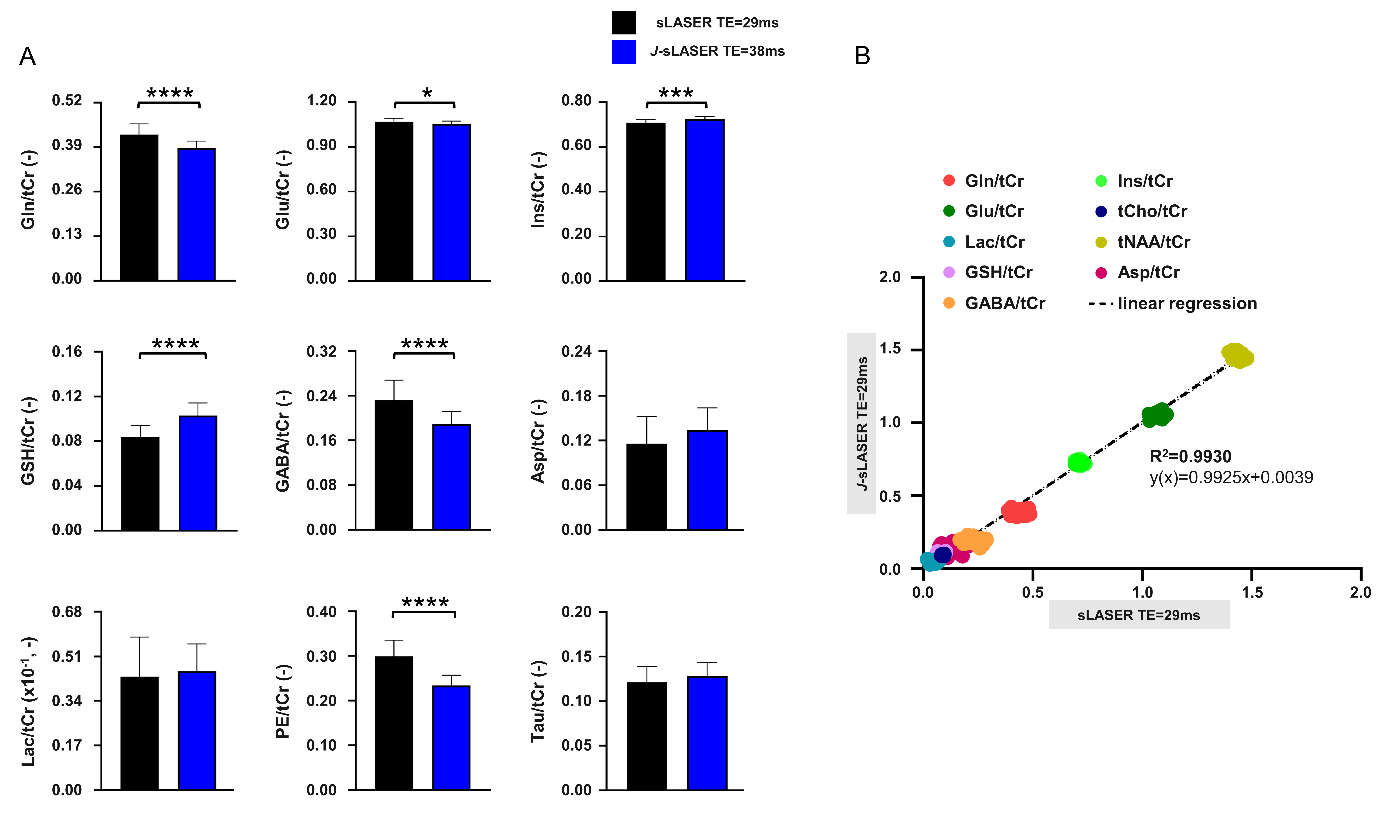
Figure S2:*** *(A) Concentrations normalized to the concentration of tCr and corrected for T2 relaxation for the shortest TE sLASER (black) and J-sLASER (blue) at TE=38ms sequences. (B) Correlation between concentration ratios of each metabolites (one color per metabolite) for both sequences, showing almost a 1:1 relation. Statistical significance between both sequences (represented with * were evaluated using a unpaired t- test *p<0.05, **p<0.005, ***p < 0.001, ****p ≤ 0.0001.*

*
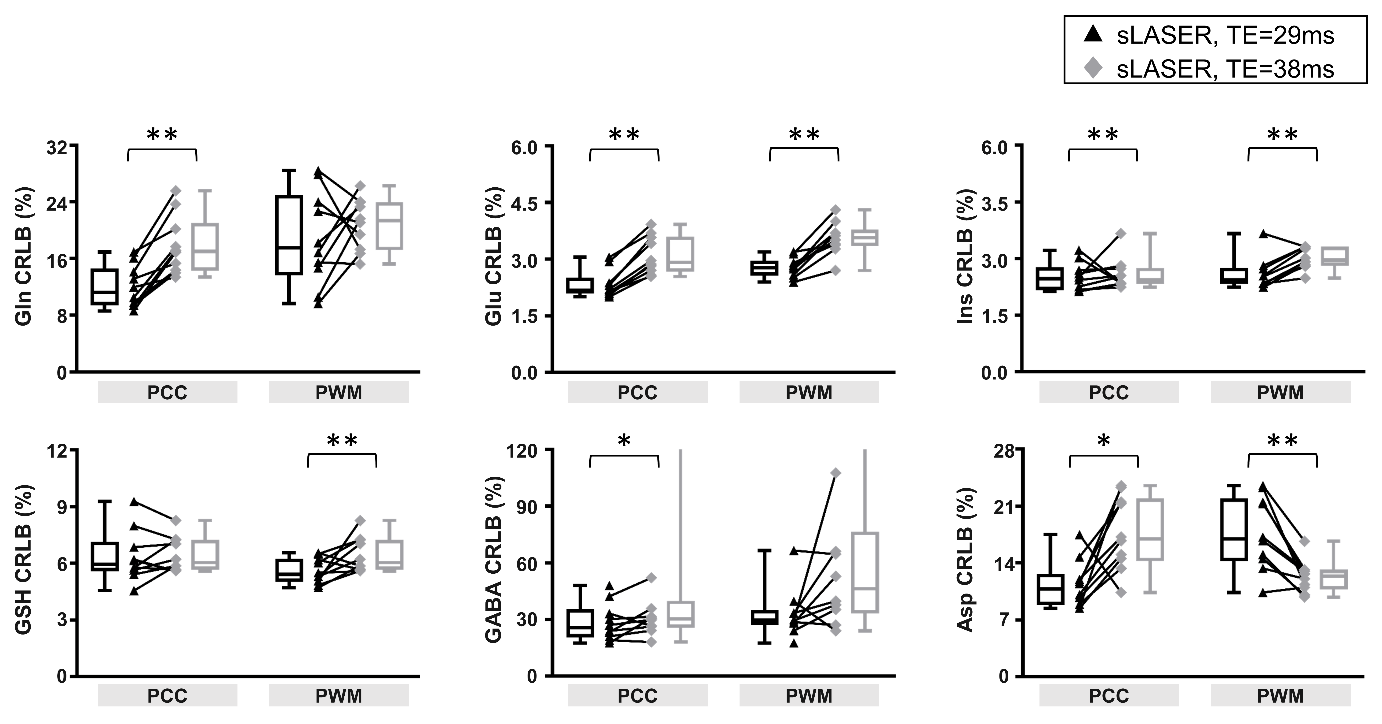
****Figure S3:*** *CRLB values estimated for several J-coupled metabolites from sLASER acquisition at shortest TE and TE=38ms in both regions are represented. Whisker plots (min-max) and individual data are shown. The black triangle represents the sLASER at TE=29ms individual data and the gray diamond represents the sLASER at TE=38ms individual data. The black line connect data from same individual. Statistical significance between both sequences (represented with *) were evaluated using a Wilcoxon signed-rank paired test with *p<0.05 and **p<0.005.*

***Figure S4:*** *Data acquired with sLASER and J-sLASER sequences in PCC from the cross-sectional and repeatability studies were combined. CRLB values estimated for several J-coupled metabolites are displayed. Whisker plots (min-max) and individual data are shown. The black triangle represents the sLASER individual data and the blue diamond represents the J-sLASER individual data. The black line connects data from same individual. Statistical significance between sLASER and J-sLASER (represented with *) were evaluated using a Wilcoxon signed-rank paired test with *p<0.05 and **p<0.005.*

***
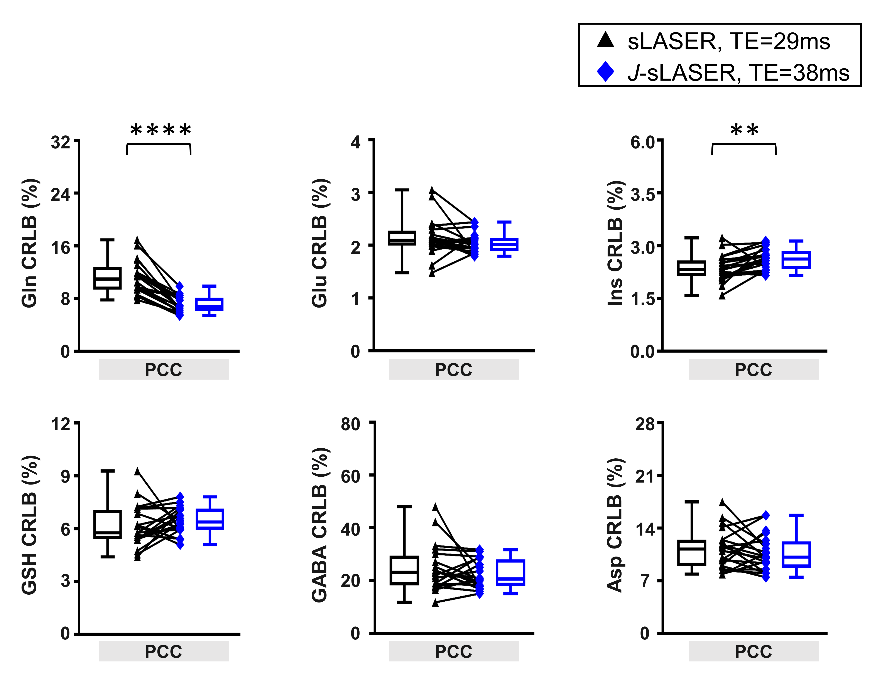
***


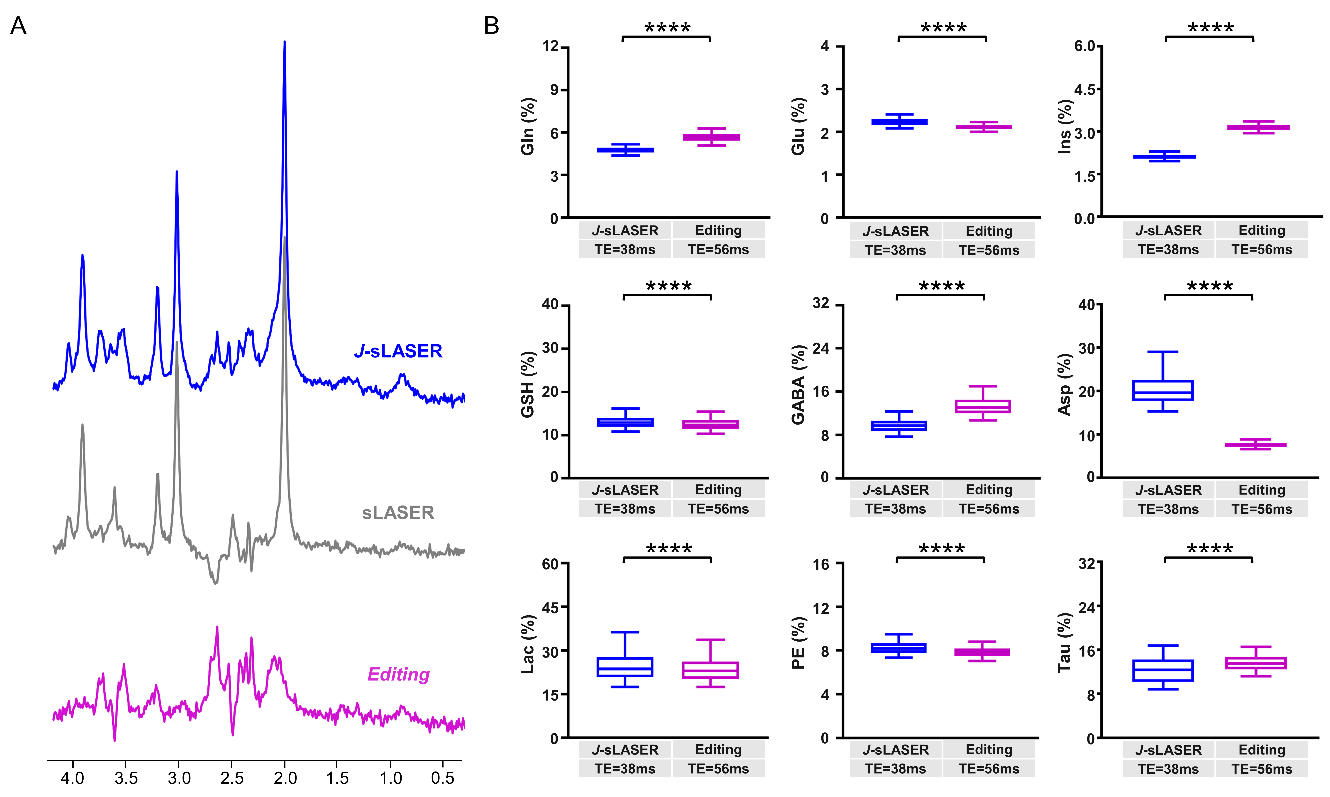
***Figure S5:*** *(A) Representative ‘brain-like’ spectra simulated for sLASER, J-sLASER and edited (subtraction of J-sLASER and sLASER) at TE=56ms. (B) Whisker plots (5-95 percentile) representing the distributions of the LCModel CRLB values (%) over 1000 repetitions for both the J-sLASER at TE=38ms and edited-J-sLASER at TE=56ms.*

***
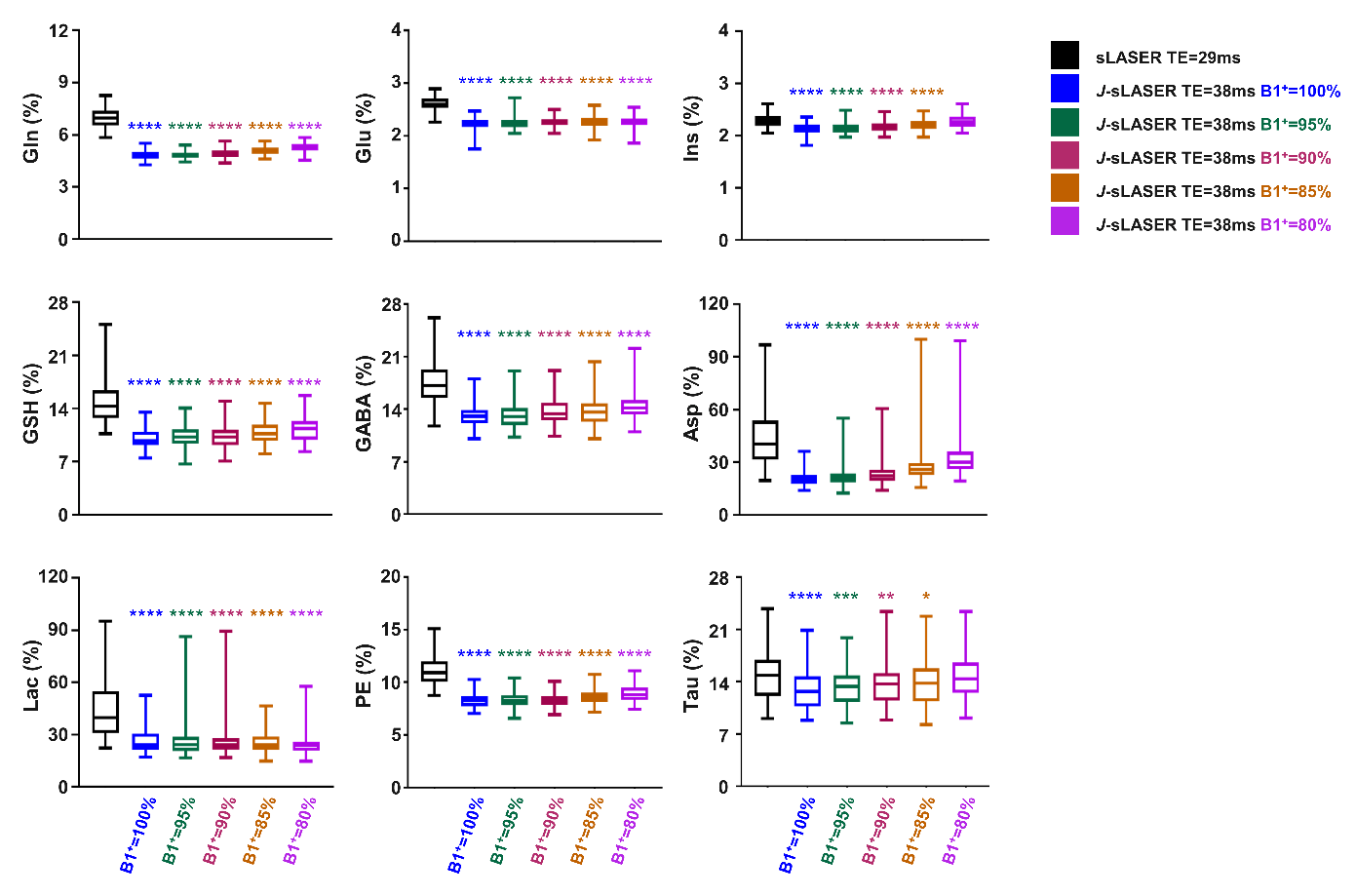
Figure S6:*** *We explored the effect of deviations from the nominal flip angle of the π/2 square RF pulse applied in the J-sLASER sequence (due to B1^+^ inhomogeneities). We simulated 100 ‘brain-like’ spectra for the J-sLASER sequence at TE=38ms with 5 scaling factors for the π/2 square RF pulse: 0.8, 0.85, 0.9, 0.95, and 1 (resulting in the following flip angles: 72º, 76.5º, 81º, 85.5º, and 90º). We compared the CRLBs measured under these conditions to the CRLBs estimated from 100 ‘brain-like’ spectra generated with the sLASER sequence at TE=29ms. Whisker plots (min-max) represent the distributions of the LCModel CRLB values (%) over 100 repetitions. CRLB values above 100 were discarded here, resulting in removal of 5 data points for Asp for the sLASER sequence at TE=29ms. An unpaired Student’s t test with equal variance (GraphPad Software, USA) was used to compare each J-sLASER acquisition to the sLASER acquisition. A threshold of p < 0.05 was considered significant, the following symbols where used to indicate the significance: *p < 0.05, **p < 0.01, ***p < 0.001, ****p ≤ 0.0001. Except CRLB(Tau) and CRLB(Ins) at the lowest scaling factor (0.8), CRLB values measured for all J-sLASER conditions were significantly lower than when measured using sLASER data.*
